# Maternal care boosted by paternal imprinting in mammals

**DOI:** 10.1371/journal.pbio.2006599

**Published:** 2018-07-31

**Authors:** H. D. J. Creeth, G. I. McNamara, S. J. Tunster, R. Boque-Sastre, B. Allen, L. Sumption, J. B. Eddy, A. R. Isles, R. M. John

**Affiliations:** 1 Biomedicine Division, School of Biosciences, Cardiff University, Cardiff, United Kingdom; 2 Behavioural Genetics Group, MRC Centre for Neuropsychiatric Genetics and Genomics, Neuroscience and Mental Health Research Institute, Cardiff University, Cardiff, United Kingdom; Harvard University, United States of America

## Abstract

In mammals, mothers are the primary caregiver, programmed, in part, by hormones produced during pregnancy. High-quality maternal care is essential for the survival and lifelong health of offspring. We previously showed that the paternally silenced imprinted gene *pleckstrin homology-like domain family A member 2* (*Phlda2*) functions to negatively regulate a single lineage in the mouse placenta called the spongiotrophoblast, a major source of hormones in pregnancy. Consequently, the offspring’s *Phlda2* gene dosage may influence the quality of care provided by the mother. Here, we show that wild-type (WT) female mice exposed to offspring with three different doses of the maternally expressed *Phlda2* gene—two active alleles, one active allele (the extant state), and loss of function—show changes in the maternal hypothalamus and hippocampus during pregnancy, regions important for maternal-care behaviour. After birth, WT dams exposed in utero to offspring with the highest *Phlda2* dose exhibit decreased nursing and grooming of pups and increased focus on nest building. Conversely, ‘paternalised’ dams, exposed to the lowest *Phlda2* dose, showed increased nurturing of their pups, increased self-directed behaviour, and a decreased focus on nest building, behaviour that was robustly maintained in the absence of genetically modified pups. This work raises the intriguing possibility that imprinting of *Phlda2* contributed to increased maternal care during the evolution of mammals.

## Author summary

Female mammals are primed during pregnancy for their new role as a mother caring for their newborn. Indirect evidence suggests that this behaviour is, in part, instructed by hormones produced by the foetally derived placenta. We previously reported that the Phlda2 gene controls the size of the placental endocrine compartment that produces hormones. Phlda2 is subject to the remarkable epigenetic process called genomic imprinting, in which one parental allele is switched off. In the case of Phlda2, this is the paternal allele. This raises the intriguing possibility that the father’s genome influences the quality of care that offspring receive from their mothers. Here, we show via genetic manipulation of pup embryos that female mice pregnant with pups carrying the highest dose of Phlda2 favour nest building over caring for their young or themselves. In contrast, dams exposed to the lowest Phlda2 dose while pregnant prioritise nurturing and grooming of their young and personal grooming. These changes in maternal focus suggest the possibility that imprinting of Phlda2 contributed to enhanced maternal care in mammals. This study also presents a unique example of how the genetic makeup of offspring can influence maternal behaviour.

## Introduction

High-quality maternal care is vitally important for newborn survival and their behavioural and metabolic health later in life, evidenced by the catastrophic consequences when maternal care is poor or absent [1–3] (S1 Table). Maternal care provision comes at the cost of the mother’s later reproductive fitness, whereas the paternal interest is best served by prolonged care of progeny exclusively by the dam. Imprinted genes, expressed from a single parental allele as a consequence of germline epigenetic events [4], are thought to be a physical embodiment of this conflict between male and female mammals over maternal investment in offspring [5, 6]. Essentially, ‘paternalisation’ (silencing of paternal allele) is proposed to secure higher maternal investment, while ‘maternalisation’ counteracts to protect maternal reproductive fitness [5]. While there are further refinements, alternative hypotheses, and much debate [6–8], most theories are consistent with the existence of imprinted genes that influence maternal care provision. Disruption of the expression of three imprinted genes in the mother (*paternally expressed gene 1* [*Peg1*], *paternally expressed gene 3* [*Peg3*], and *type 3 deiodinase*) results in deficits in maternal care [9–11], and we have recently shown that loss of *Peg3* in the offspring increases maternal anxiety and decreases pup retrieval in wild-type (WT) mothers [12]. However, deficits in maternal care are relatively common and have been reported with a myriad of genetic modifications as well as exposures to adverse environments in pregnancy (S1 Table). In contrast, examples of increased maternal care providing more compelling evidence for a purposeful phenomenon are exceptionally rare and almost invariably involve hormonal manipulations.

The placenta is a foetally derived organ fundamental to pregnancy [13, 14]. A number of hormones produced by, or dependent on, the placenta are implicated in the programming of maternal care during pregnancy [15] (S1 Fig). Placental lactogens, produced by placental endocrine lineages in contact with the maternal circulation [16], are known to accumulate in cerebrospinal fluid during pregnancy [17] and can stimulate maternal behaviour when infused directly into the medial preoptic area in nonpregnant rodents [18]. Placental lactogens are related to the pituitary hormone prolactin required for maternal behaviour [19, 20], and a subset bind and activate the prolactin receptor [21] also required for maternal care [22, 23]. In addition, whereas not all the genes encoding steroidogenic enzymes are expressed in the mature mouse placenta (S1 Fig), lactogenic hormones support the production of oestrogens and progesterone from the corpus luteum in rodents [24], which act in concert in the rapid induction of maternal behaviour at term [25, 26]. Together, these data strongly suggest that placental lactogens have a direct effect on maternal care behaviour. The neurotransmitter serotonin, important in maternal care [27], is manufactured by the placenta, although thought to be directed at the foetal brain [28–31]. There is less evidence that dopamine [32–34], oxytocin [35–37], or vasopressin [38, 39] could be influenced by placental genes (S1 Fig).

Using genetic mouse models, we identified several paternally silenced, maternally expressed imprinted genes that function to restrain the placental lineages that produce hormones thought to be important for maternal care behaviour in mice [40–42]. This led us to hypothesise that the silencing of one or more genes by the paternal genome may have acted to boost maternal care in mammals. The mouse *pleckstrin homology-like domain family A member 2* (*Phlda2*) gene negatively regulates the expansion of a single placental endocrine lineage called the spongiotrophoblast, a lineage that expresses placental lactogens, pregnancy-specific glycoproteins, and a number of other hormones that induce and maintain the physiological adaptations required for a successful pregnancy [16], [41, 43–46]. *Phlda2* is expressed from the maternal allele primarily in the placenta [47, 48] and encodes a pleckstrin homology (PH) domain–only protein [49] that functions to inhibit cell proliferation by repressing AKT activation [50]. Early in mouse placental development, *Phlda2* supresses the proliferation of the spongiotrophoblast [51]. Doubling the *Phlda2* gene dosage (potentially resembling the ancient preimprinted state) by means of a bacterial artificial chromosome (BAC) transgene reduces the contribution of the spongiotrophoblast lineage to the mature placenta by approximately 50%, whereas loss of expression by knockout (KO) of the maternal allele results in a 2-fold expansion of this lineage [41, 52–54]. *Phlda2* is consequently a rheostat for placental hormones expressed from, or dependent on, the spongiotrophoblast, with ‘paternalisation’ increasing their expression and ‘maternalisation’ decreasing expression.

To ask whether expression of *Phlda2* in the offspring influenced maternal behaviour, we exposed dams to three different doses of offspring *Phlda2*, excluding the confounder of genetically modified dams by using recipient transfer of embryos exclusively into WT females. All experimental dams were generated by recipient transfer to control for any effect of this manipulation on gene expression, and litters of comparable size were used throughout. We observed changes in the maternal brain that preceded exposure to the pups and altered maternal behaviour after birth, consistent with our original hypothesis.

## Results

### Prenatal gene expression

**Fig 1:**
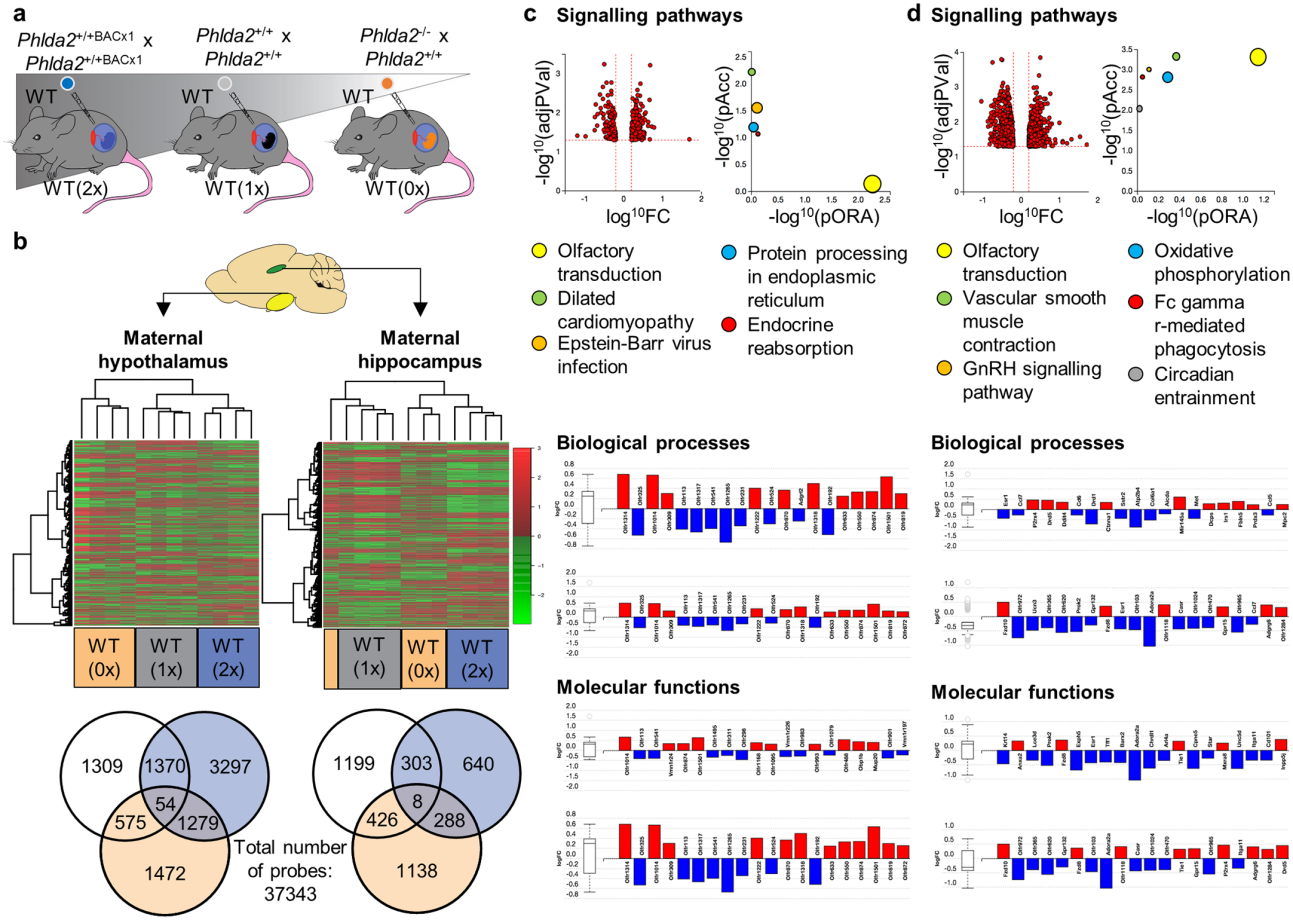
Gene alteration of prenatal maternal hypothalamus and hippocampus exposed to 0, 1, or 2 doses of offspring *Phlda2*. (A) Schematic for tissue generation. WT maternal brain tissues from *n* = 3–4 dams exposed to decreasing doses of offspring *Phlda2* were collected on E16.5 of pregnancy. (B) Gene expression data generated using the Affymetrix GeneChip platform. Three-way heat map showing differential gene expression levels between the cohorts for the hypothalamus (left panel) and hippocampus (right panel; >0.05 significance). Venn diagram from Limma analysis of significantly altered probes below. (C) Top left panel shows pathways’ perturbation versus overrepresentation for WT(2x) versus WT(0x) hypothalamus. Top right panel shows top-5 pathways plotted by evidence computed by iPathwayGuide: overrepresentation on x-axis (labelled ‘pORA’) and total pathway accumulation on y-axis (labelled ‘pAcc’). Each pathway is represented by coloured dot, with the size proportional to the size of the pathway represented. *P* values are shown as negative log (base 10) values. Biological processes and molecular functions: bar plots of top-20 genes out of the total number of differentially expressed genes. Up-regulated in red, down-regulated in blue. Box and whisker plots summarise distribution of differentially expressed genes annotated to the GO term. Box represents the first quartile, the median, and the third quartile; outliers are represented by circles. (D) Pathway analysis for WT(2x) versus WT(0x) hippocampus as described above. Additional data in S2 and S3 Figs. Raw data accession number: GSE115276. E, embryonic day; GO, gene ontology; *Phlda2*, *pleckstrin homology-like domain family A member 2*; WT, wild-type; WT(0x), WT dams carrying and caring for all *Phlda2*^−/+^ embryos (maternal inheritance of targeted allele, *Phlda2* loss of function); WT(1x), WT dams carrying and caring for all WT embryos; WT(2x), WT dams carrying >60% *Phlda2*^+/+BACx1^ embryos generated by crossing *Phlda2*^+/+BACx1^ dams and *Phlda2*^+/+BACx1^ studs.

Before undertaking a behavioural assessment, we asked whether changes were present in the maternal brain during pregnancy. Preimplantation *Phlda2*^+/+BACx1^ (BAC transgenic overexpression; 2x), *Phlda2*^*+/+*^ (wild-type; 1x), and *Phlda2*^*−/+*^ (loss of function; 0x) embryos were transferred into WT recipient females to generate WT(2x) (WT dams carrying and caring for >60% *Phlda2*^+/+BACx1^ embryos), WT(1x) (WT dams carrying and caring for all WT embryos), and WT(0x) (WT dams carrying and caring for all *Phlda2*^−/+^ embryos) dams, respectively (Fig 1A). After normalising for the number of foetuses per dam across the three groups (S2 Table), and consistent with our previous data from naturally mated litters [41, 54], foetal growth was reduced at E16.5 for both *Phlda2*^+/+BACx1^ (2x) and *Phlda2*^*-/+*^ (0x) foetuses carried by WT dams in comparison to fully WT foetuses carried by WT dams (S3a Fig; S2 Table). Gene expression in the maternal hypothalamus (onset, maintenance, and regulation of maternal behaviour) and the hippocampus (memory, learning, and responses to fear and stress) [55] was assessed at embryonic day (E) 16.5, 4 days before parturition at a time of maximal placental phenotype [41, 54]. Heat maps were plotted for the genes that showed changes in expression levels at *p* < 0.05. This resulted in a dendrogram grouping the WT(2x), WT(1x), and WT(0x) gene profiles by foetal *Phlda2* dosage (Fig 1B). Two hundred ninety-six probes (0.79%) were altered between WT(0x) and WT(2x) hypothalamus, and 1,333 probes (3.6%) were altered between WT(0x) and WT(2x) hippocampus. Analysis of hypothalamic and hippocampal pathways identified disturbances in olfactory transduction pathways important for maternal care and G protein–coupled receptor (GPCR) pathways through which neuropeptides and hormones mediate their action (Fig 1C and 1D; S3 Fig). Specific to the hippocampus were disturbances in the gonadotropin-releasing hormone signalling pathway, cell differentiation, and circadian entrainment (Fig 1D). Together, these findings indicated a physiological response to the placental cues with potential for a behavioural response.

**Fig 2:**
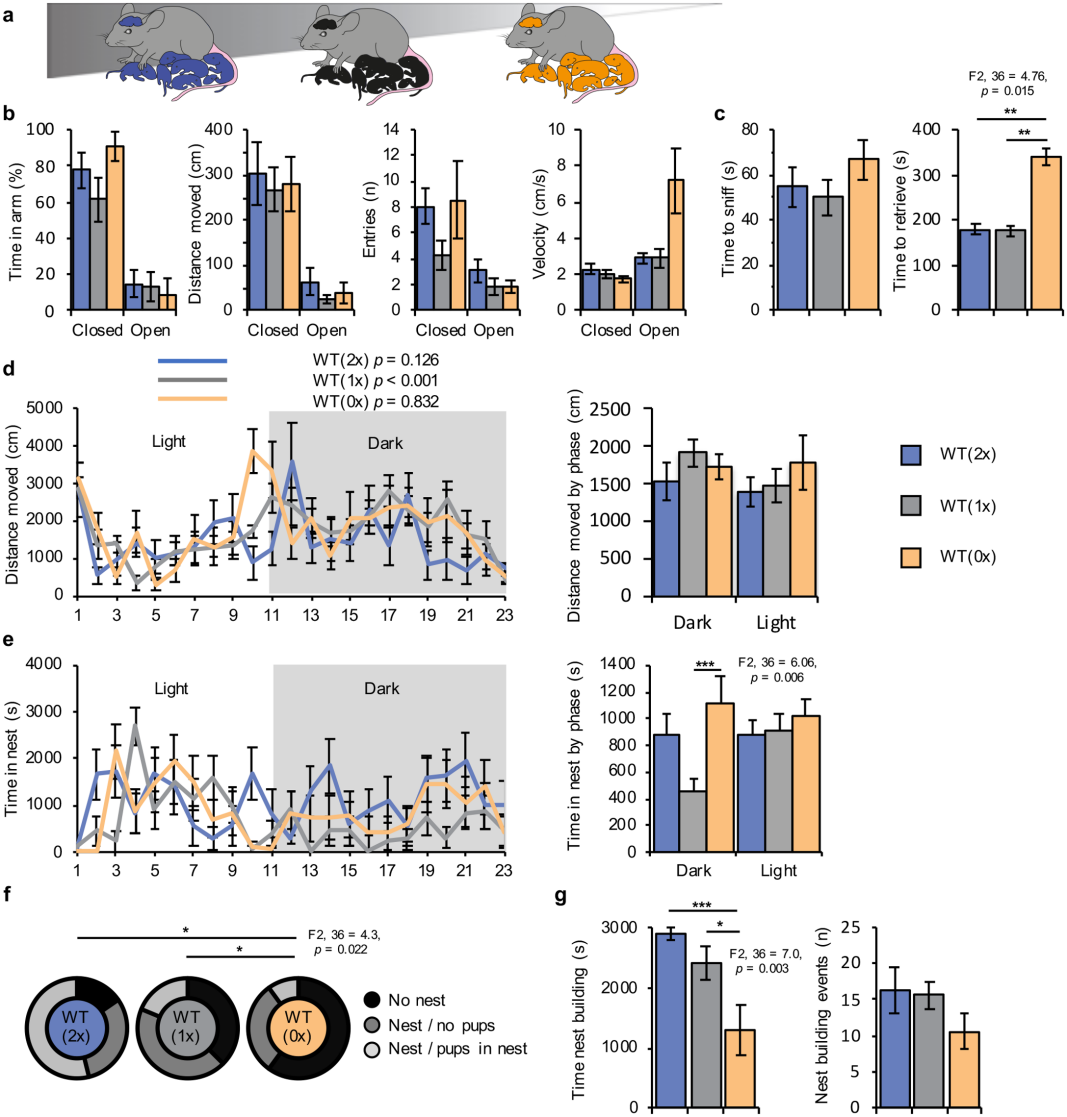
Classic tests of maternal behaviour reveal altered behaviour in WT dams exposed to different doses of offspring *Phlda2*. (A) Schematic of behavioural paradigm. WT(0x) *n* = 13, WT(1x) *n* = 14, and WT(2x) *n* = 11 experimental dams carrying and caring for pups with 0, 1, or 2 active copies of *Phlda2*. (B) EPM performed on P2. Percentage of time spent in open and closed arms, number of entries in the open and closed arms, distance moved, and mean velocity measured over 5 minutes. (C) Pup retrieval task on P3. Time taken to sniff and retrieve the first pup measured over 20 minutes. (D) Activity tracking during a 23-hour period after pup-retrieval task. Distance travelled per hour during each phase and total distance travelled during the light phase and dark phases. (E) Time spent on the nest over 23 hours and total time spent in nest during the light phase and dark phase. (F) Nest building task on P4. Dams scored on their ability to build a nest during a 1-hour test (no nest built; nest built/no pups moved into nest; nest built/pups moved into nest). Of the dams, 7/11 WT(2x), 3/14 WT(1x), and 1/13 WT(0x) built a nest and placed at least 1 pup inside within the time limit. (G) Time spent building nest (left panel) and number of nest building events (right panel). Numerical data can be found at https://osf.io/543jg/ “RAW NUMERICAL DATA.xlsx”, Sheets labelled EPM, Pup_Retrieval_Nest_Building, Ethovision_23hr. Data shown as mean ± SEM. Significance determined using one-way ANOVA Dunnet post hoc test. Statistical significance: *p < 0.05, **p < 0.01, and ***p < 0.005. EPM, elevated plus maze; P, postnatal day; *Phlda2*, *pleckstrin homology-like domain family A member 2*; WT, wild-type; WT(0x), WT dams carrying and caring for all *Phlda2*^−/+^ embryos (maternal inheritance of targeted allele, *Phlda2* loss of function); WT(1x), WT dams carrying and caring for all WT embryos; WT(2x), WT dams carrying >60% *Phlda2*^+/+BACx1^ embryos generated by crossing *Phlda2*^+/+BACx1^ dams and *Phlda2*^+/+BACx1^ studs.

Following these encouraging findings, a second cohort of dams was generated by the same protocol (Fig 2A) and allowed to deliver. As for the gene expression analysis, dams with small litters (<6) were excluded to generate comparable litter sizes across the three groups (F2, 36 = 0.83, *p* = 0.45; S2 Table; S2 Fig). Behaviour was assessed concurrently by the same team of researchers between postnatal day (P) 2 and P4, when maternal behaviours are most intense in rodents [56, 57].

To assess anxiety behaviour, we made use of the elevated plus maze (EPM) test on P2 (Fig 2A). Duration spent within the open and closed zones (F2, 36 = 1.44, *p* = 0.25) and frequency of entry into each arm (F2, 36 = 1.7, *p* = 0.19) did not differ between cohorts. There was no difference in time spent in the most anxiogenic open zone (F2, 36 = 0.16, *p* = 0.85). The total distance moved within each zone was the same (F2, 36 = 1.58, *p* = 0.22), and there was no difference between the speed of travel of the dams across the different zones (F2, 36 = 2.87, *p* = 0.069). There were no differences in rearing (duration or frequency), stretch-attend (duration or frequency), or head-dips (duration or frequency) in any zone (data in S2 Table).

On P3, dams were tested on the pup-retrieval task after 24-hour acclimatisation in Phenotyper cages. The time taken by dams to first sniff their pups did not vary with *Phlda2* dosage (F2, 36 = 1.1, *p* = 0.33; Fig 2C), indicating intact response to olfactory cues. However, the time taken to retrieve the first pup was different between the three groups (F2, 36 = 4.8, *p* = 0.015; Fig 2C). WT(0x) dams who carried and cared for offspring with the lowest expression of *Phlda2* took 3 times longer to retrieve their first pup compared to either WT(1x) dams (*p* = 0.01) or WT(2x) dams (*p* = 0.01; additional data in S2 Table).

### Nocturnal behaviour

Lactating dams are usually more active during the dark phase [58], which was observed with the WT(1x) dams exposed to the normal dose of *Phlda2* (*p* < 0.001; Fig 2D). In contrast, neither WT(0x) (*p* = 0.83) nor WT(2x) (*p* = 0.13) dams showed a difference in activity between light and dark phases. Although all three travelled similar distances over a 23-hour period (36,022 versus 39,191 versus 39,542; WT[2x] versus WT[1x] versus WT[0x]; F2, 36 = 5.1, *p* = 0.608), WT(0x) dams showed a tendency to be more active during the day, whereas WT(2x) dams were less active at night (Fig 2D). The time dams spent in the nest during the light phases was similar across the three cohorts (F2, 36 = 0.29, *p* = 0.75), but during the dark phase (F1, 25 = 5.8, *p* = 0.024; Fig 2E), WT(0x) dams spent more time in the nest compared to WT(1x) dams (*p* = 0.005; Fig 2E).

### Nest building

Dams showed differences in nest building behaviour (F2,36 = 4.3, *p* = 0.022; Fig 2F). On P4, WT(2x) dams were more effective at making nests and placing at least 1 pup in the nest than either WT(1x) (*p* = 0.05) or WT(0x) dams (*p* = 0.03). WT(0x) dams were least effective in this task, with only 1 WT(0x) dam building a nest and placing at least 1 pup within the nest during the 60-minute test. Consistent with success rate in this task, there were differences across the cohorts in time spent building nests (F2, 36 = 6.967, *p* = 0.003; Fig 2G), with WT(0x) dams spending less time building nests than either WT(1x) (*p* = 0.029) or WT(2x) dams (*p* = 0.002).

Isolation-induced ultrasonic vocalisations (USVs) are known to stimulate maternal behaviours such as nest building and pup retrieval [59, 60]. We found no difference between *Phlda2*^+/+^ and *Phlda2*^−/+^ (KO) pup vocalisation at P2 or P4 that might explain delayed retrieval or the failure to build nests (S4 Fig; data from naturally mated litters).

### Nursing and grooming

**Fig 3:**
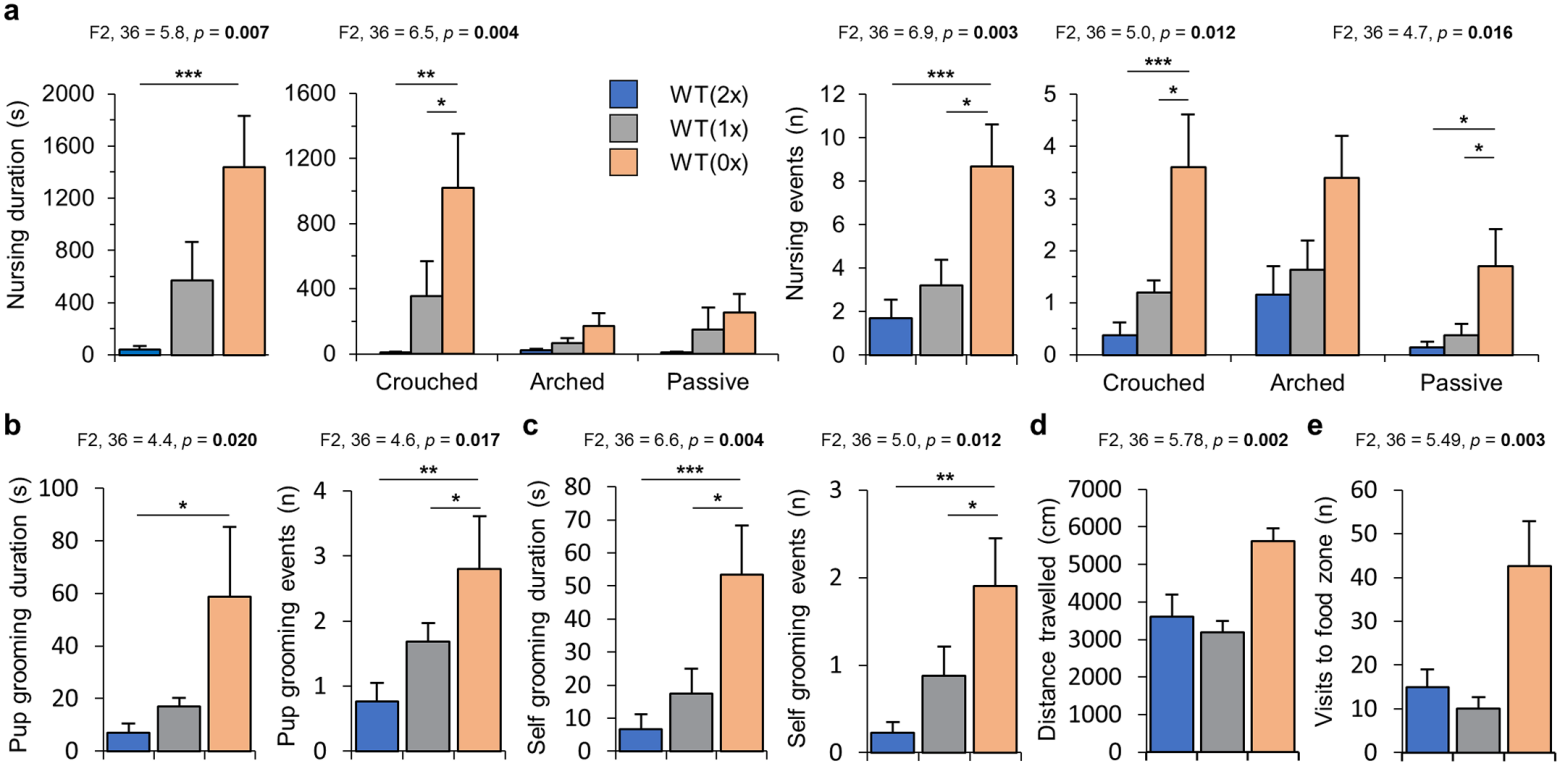
Alterations in priorities of ‘paternalised (0x)’ and ‘maternalised (2x)’ dams during the nest building task. (A) Nursing behaviours during the 1-hour nest building task. Duration of nursing (left panels) presented as total nursing duration and duration of crouched, arched, or passive nursing. Number of pup nursing events (right panels) presented as total events and by each category. (B) Duration spent grooming pups (left panel) and number of pup grooming events (right panel). (C) Duration spent self-grooming (left panel) and number of self-grooming events (right panel). (D) Ethovision tracking of distance travelled by dams during the nest building task. (E) Number of visits to the food zone during the nest building task. Additional data in S2 Table. Numerical data can be found at https://osf.io/543jg/ “RAW NUMERICAL DATA.xlsx”, Sheets labelled Nest_Building_Behaviour. Data are shown as mean ± SEM. Significance determined using one-way ANOVA Dunnet post hoc test. Statistical significance: *p < 0.05, **p < 0.01, and ***p < 0.005. WT(0x), WT dams carrying and caring for all *Phlda2*^−/+^ embryos (maternal inheritance of targeted allele, *Phlda2* loss of function); WT(1x), WT dams carrying and caring for all WT embryos; WT(2x), WT dams carrying >60% *Phlda2*^+/+BACx1^ embryos generated by crossing *Phlda2*^+/+BACx1^ dams and *Phlda2*^+/+BACx1^ studs.

Pup retrieval and nest building are classic tests used to assess maternal behaviour but are not necessarily indicators of enhanced maternal care. Dams normally divide their time between nursing and grooming their pups (pup focused), maintaining themselves (self-directed), and nondirected maternal behaviours (protection of the young, nest building). During the nest building task, there is a conflict between these essential behaviours. Consistent with this conflict, during the task, there were differences in the duration and frequency of pup nursing (F2, 36 = 5.8, *p* = 0.007 and F2, 36 = 6.9, *p* = 0.003; Fig 3A). Dams exposed to the lowest dose of *Phlda2* (0x) engaged in crouched nursing for longer than dams exposed to the highest dose (WT[0x] versus WT[2x]; *p* = 0.005 and *p* = 0.003; Fig 3A). The number of crouched nursing events was greater for WT(0x) females, as was the number of passive nursing events (Fig 3A).

Both the duration (F2, 36 = 4.39, *p* = 0.02) and frequency (F2, 36 = 4.59, *p* = 0.017) of pup grooming events was also different between the three groups (Fig 3B). WT(0x) dams spent 5 times longer grooming their pups (*p* = 0.02) and engaged in twice the number of grooming events (*p* = 0.012) than WT(2x) dams. In addition to changes in pup-directed behaviour, dams differed in both the duration (F2, 36 = 6.61, *p* = 0.004) and frequency (F2, 36 = 4.4, *p* = 0.02) of self-grooming (Fig 3C). WT(0x) spent longer than either WT(1x) (*p* = 0.021) or WT(2x) dams (*p* = 0.003) in self-grooming and engaged in self-grooming more frequently (*p* = 0.02 and *p* = 0.01, respectively). WT(0x) dams travelled further during the nest building task (Fig 3D) and visited the food zone more often during the nest building task than dams exposed to higher doses of *Phlda2* (Fig 3E). Additional data are in S2 Table. As the dosage of *Phlda2* decreased in the offspring, dams spent more time caring for their pups and maintaining themselves and less time on the ‘housekeeping’ task.

### Pup welfare

Pup growth can be regarded as a measure of maternal care correlating with time spent nursing and lactation. *Phlda2*^+/+BACx1^ and *Phlda2*^*−/+*^ foetuses were approximately 16% lighter than *Phlda2*^*+/+*^ foetuses at E16.5 (S2 Fig). Pups were not weighed at birth to avoid disturbing the dams in this critical period, but by P7, there was no difference in pup weight between the three cohorts (F2, 164 = 1.89, *p* = 0.15; S2 Table). Adequate preweaning weight gain indicated that the altered maternal behaviour did not negatively impact pup welfare, at least in the short term.

### Prenatal programming versus postnatal stimulation

**Fig 4:**
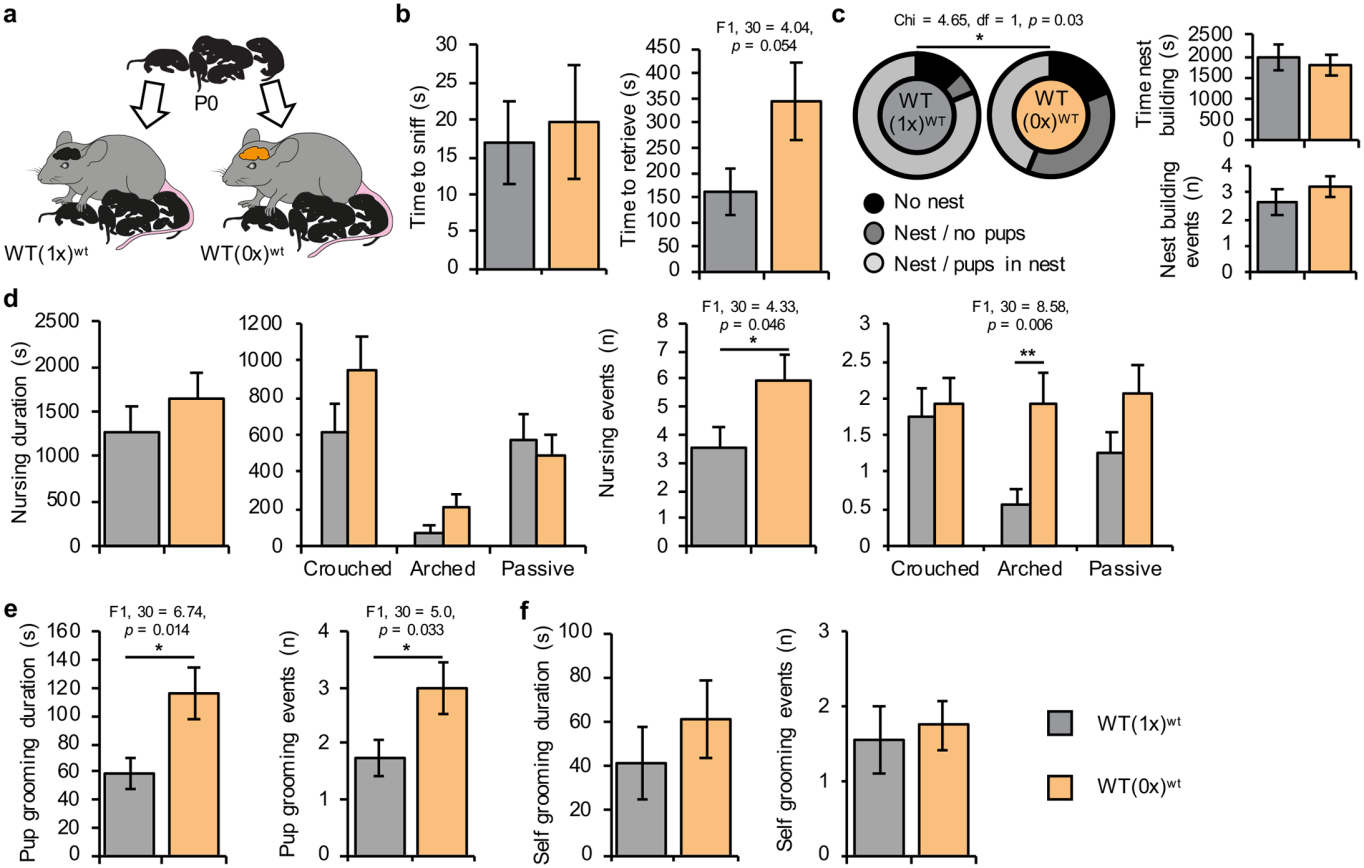
Increased maternal care by ‘paternalised’ (0x) dams maintained in the absence of cues from *Phlda2*^−/+^ (KO) pups. (A) Schematic of behavioural paradigm. WT(1x) and WT(0x) dams were generated by recipient transfer. On day of delivery, WT pups born from WT(1x) dams were fostered to nonparental WT(1x) dams or WT(0x) dams to generate WT(1x)^wt^ (*n* = 15) and WT(0x)^wt^ (*n* = 15) cohorts and left for 3 days to acclimatise in the home cage. (B) Pup retrieval in home cage on P3. Time taken to sniff (left) and time taken to retrieve first pup (right). (C) Nest quality (left), duration of nest building (middle), and nest building events (right) scored on P4. Dams were scored based on their ability to build a nest during a 1-hour test. Seven out of 15 WT(0x)^wt^ dams built nests and placed fostered pups inside within 60 minutes, compared to 13 out of 15 WT(1x)^wt^ dams. (D) Duration and number of total nursing events (left) and by type of nursing (right) during 60-minute nest building task. (E) Duration (top) and number (bottom) of pup grooming events during 60-minute nest building task. (F) Duration and number self-grooming events during 60-minute nest building task. Additional data in S3 Table. Numerical data can be found at https://osf.io/543jg/ “RAW NUMERICAL DATA.xlsx”, Sheets labelled CrossFostering, CrossFostering_Retrieval. Data shown as mean ± SEM. Significance determined using one-way ANOVA. KO, knockout; P, postnatal day; *Phlda2*, *pleckstrin homology-like domain family A member 2*; WT, wild-type; WT(0x), WT dams carrying and caring for all *Phlda2*^−/+^ embryos (maternal inheritance of targeted allele, *Phlda2* loss of function); WT(0x)^wt^, WT dams carrying all *Phlda2*^−/+^ embryos and caring for all WT pups; WT(1x), WT dams carrying and caring for all WT embryos; WT(1x)^wt^, WT dams carrying all WT embryos and caring for all WT pups from a different WT litter).

Our understanding of the function of *Phlda2* in placental endocrine lineage development alongside alterations in the maternal brain that preceded exposure to the pups suggested the prenatal programming of the behavioural changes. To test this theory, WT dams exposed in utero to *Phlda2*^*−/+*^ (1x) or *Phlda2*^*−/+*^ (0x) embryos were provided with fully WT pups immediately after birth (Fig 4A), followed by an assessment of behaviours previously shown to be altered in WT(0x) dams. Delayed retrieval was not different between the two groups (Fig 4B). WT(0x)^wt^ dams (WT dams carrying all *Phlda2*^−/+^ embryos and caring for all WT pups) were less effective at nest building than WT(1x)^wt^ dams (WT dams carrying all WT embryos and caring for all WT pups from a different WT litter), despite a similar duration and number of events (Fig 4C). Total number of nursing events was greater for WT(0x)^wt^ dams (F1, 30 = 4.33, *p* = 0.046), with arched nursing events showing the greatest difference between the two groups (F1, 30 = 8.58, *p* = 0.006) (Fig 4D). Both the duration and number of pup grooming events by WT(0x)^wt^ dams were increased relative to WT(1x)^wt^ dams (F1, 30 = 6.74, *p* = 0.014 and F1, 30 = 5.0, *p* = 0.033; Fig 4E). Self-grooming behaviour was similar between the two groups (Fig 4F). Additional data are in S3 Table.

Despite the significant disruption imposed by removing their own newborns and replacing them with another dam’s newborns on the day of birth, WT(0x)^wt^ dams retained their enhanced nursing and grooming behaviours 4 days later. These data support prenatal programming of at least some aspects of the maternal care phenotype. Moreover, replicating our findings from the original experiment demonstrates that the phenotype is robustly reproducible.

## Discussion

Previous studies have shown that imprinted genes expressed in the dam are required for high-quality maternal care provision [9–11]. Loss of expression of imprinted genes in the offspring can also negatively influence maternal care postnatally through reduced demand for milk [61] or reduced communication by the pups [12]. Here, we show that the offspring can influence the maternal care they will receive even before they are born by modulating expression of the imprinted *Phlda2* gene in their placenta. Critically, this is a rare example of an alteration that elicits enhanced care from the mother, reproducible over two independent studies. The direction of imprinting (paternalisation) supports our original hypothesis that silencing of *Phlda2* in the male germline contributed to the evolution of enhanced maternal care in mammals.

In mammals, male involvement in the care of young is rare, although not unknown [62], because only females lactate, and internal fertilisation ensures maternity but not paternity. Genomic conflict over maternal care predicts that ‘paternalisation’ should result in increased maternal care, securing higher maternal investment [5]. However, this investment should not come at a substantial cost to maternal well-being, as pups are dependent on the mother until weaning. Consistent with this, we observed that ‘paternalised (0x)’ dams exposed to the lower *Phlda2* dose spent more time nursing and grooming their pups and more time on self-maintenance behaviours than maternalised (2x) dams. Indeed, it may be that paternal silencing of *Phlda2* increases the fitness of the mothers, at least in the short term while caring for her litter. Importantly, despite removing newborn pups from the nest and replacing them with WT pups, paternalised (0x) dams retained their enhanced nurturing behaviour in the absence of continued exposure to genetically modified offspring 4 days later. Not only does this finding support a prenatal mechanism, our ability to replicate findings at a different time and under considerably more disruptive circumstances demonstrates the robustness of the phenotype. In contrast, WT(2x) dams exposed to the higher dose of *Phlda2* in their offspring spent less time grooming and nursing their pups and less time on self-grooming. In most behaviours, WT(1x) dams displayed either an intermediate phenotype or were similar to WT(2x) dams. Changes in nurturing behaviour were subtle, and the decreased nurturing observed with WT(2x) dams did not have an immediate impact on pup welfare. Following up the longer-term metabolic and behavioural outcomes for the offspring will be required to fully establish the consequences of decreased maternal care. Poor maternal care can be passed on [2, 63, 64], and it will be equally interesting to ask whether the altered behaviour persists in subsequent pregnancies.

Maternal nurturing was increased in response to the lower dose of *Phlda2*, but WT(0x) dams were slower to retrieve their first pup. Dams locate displaced pups through olfactory and auditory cues. Dams from the three groups were equally effective at locating and sniffing their pups, and *Phlda2*^*−/+*^ pups made normal USVs when separated from their mothers, arguing against a major deficit in either medium. Rat dams can selectively retrieve the best-developed pups of the litter [65]. Both *Phlda2*^*−/+*^ or *Phlda2*^*+/+BACx1*^ pups are growth restricted at birth [41, 54], and the delay in WT(0x)^wt^ dams retrieving the foster WT pups was not statistically significant. It is possible that the quality of the offspring is responsible for the observed delay in retrieval. WT(0x) females were less focused on nest building, spending a shorter duration on this task, with fewer events and with only 1/13 females successfully building a nest and placing at least 1 pup inside. In contrast, WT(2x) dams showed greater focus on nest building, spending longer on this task, with an increased number of events and 7/13 dams successfully completing the task, performing even better than WT(1x) dams. When pups are displaced from the nest, dams normally rapidly return pups [66], with both retrieval and nest building important for neonatal pup thermoregulation. Pup retrieval and nest building are classically used to assess maternal behaviour, but these behaviours per se are not necessarily an indicator of ‘good mothering’. During nest building, there is a conflict between the time the dam spends on the nest and the time spent direct nurturing her pups and herself. Although more elaborate nest building and pup retrieval are necessary aspects of maternal behaviour, one interpretation is that paternalised (0x) dams are better mothers, as they are more focused on direct nurturing.

The most simple explanation for our findings is that alterations in priming of mammary development by placental lactogens drive increased or decreased milk availability, impacting nursing behaviour with altered nest building as a secondary effect. However, dams with lactation deficits can initiate normal maternal behaviour [67, 68]. Moreover, changes in the dams’ hypothalamic and hippocampal transcriptomes were present 4 days before birth, indicating a prenatal component. The programming of maternal behaviour during pregnancy requires prolonged exposure to a number of hormones expressed from, or dependent on, the placenta. *Phlda2* plays a key role in restricting the expansion of the spongiotrophoblast, which is the major endocrine cell type of the placenta [69]. Just 2-fold expression of *Phlda2* prevents the expansion of this lineage by 50%, with a concurrent decrease in the expression of spongiotrophoblast-expressed hormones, while loss of expression results in a 200% increase in spongiotrophoblast and increased hormone expression [41]. Consistent with a direct action of hormones on the maternal brain, analysis of hypothalamic and hippocampal pathways identified alterations in GPCR pathways through which neuropeptides and hormones mediate their action. Olfactory transduction pathways important for maternal care [15, 70, 71] were altered in both the maternal hippocampus and hypothalamus. Olfaction is important for pup recognition connected to the emotional system regulating social motor behaviour, which is initiated by odour, and the way in which positive signals of rewards are valued, which are important for mother–pup bonding. The hippocampal olfactory circuit is linked to odour-guided learning and memory, potentially also influencing mother–pup bonding. The gonadotropin-releasing hormone signalling pathway altered in the hippocampus has been implicated in maternal care and is known to respond to prolactin via this receptor [72, 73]. Placental lactogens have previously been implicated in the induction of maternal care [18], potentially via an interaction with the prolactin receptor [22, 23], expressed in both the maternal hippocampus and hypothalamus [74]. Together, these data suggest changes in the expression of one or more placental lactogens contribute to the changes in behaviour we observe in our dams. In mice, there are 22 placenta-specific, Prl-related genes expressed from the trophoblast giant cells, spongiotrophoblast, and glycogen cell lineages [16, 75]. Of these, Prl3d1 (PL-I) and Prl3b1 (PL-II) have been shown formally to bind to the Prl receptor [21]. Prl3b1 is the dominant form during the second half of pregnancy [76], and expression of this hormone is significantly affected by loss and gain of the spongiotrophoblast mediated by *Phlda2* [41, 53]. However, while it seems likely that *Phlda2* acts, at least in part, on maternal behaviour via regulating the production of *Prl3b1*, given the complexity of maternal care, it is unlikely that *Phlda2* functions via a single hormone or pathway.

While we have not sought here to identify the specific hormone(s) that contributes to the prenatal programming of maternal care, we have shown that imprinted genes can overcome the rapid evolution of placental hormone gene families [77] by regulating the lineages that express these hormones rather than directly regulating individual hormones, at least in mice. The placenta is the most diverse organ in mammals [78, 79], and this phenomenon may not be conserved across species. We have previously reported that in human pregnancies, placental *PHLDA2* expression is inversely correlated with maternal serum human placental lactogen [80]. It remains to be seen if this relationship applies to other eutherian mammals.

**Fig 5:**
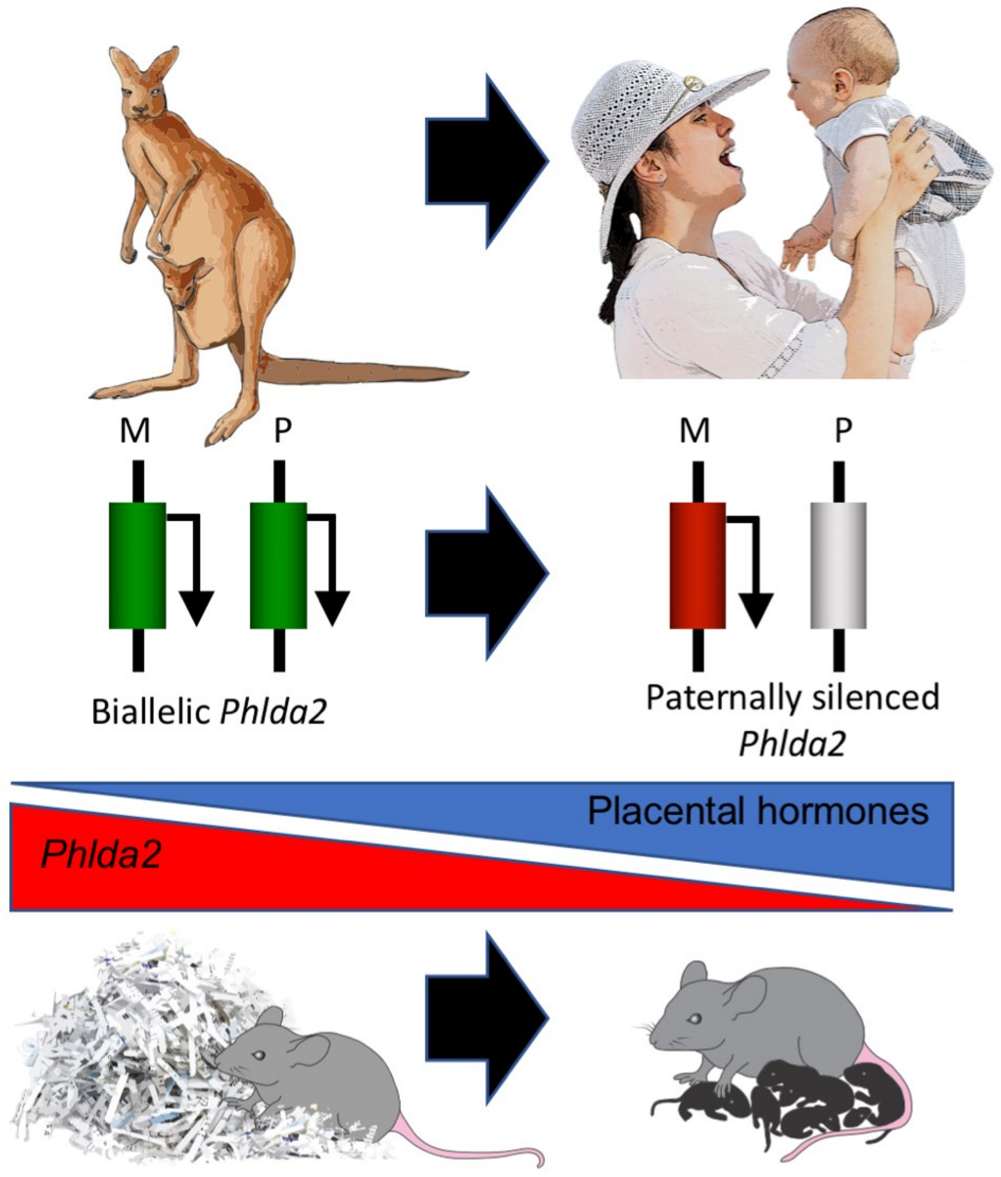
*Phlda2* imprinting may have increased maternal care provision during the evolution of mammals. *Phlda2* regulates the production of placental hormones by controlling lineage allocation in the placenta. Decreasing *Phlda2* expression through imprinting increases placental hormone production, programming the mother to increase her focus on nurturing her young. Maternal silencing of *Phlda2* occurred after marsupials diverged from eutherian mammals more than 160 million years ago. Postpartum development in marsupials predominantly takes place in a pouch, with maternal care being most prominent after the young leave the pouch. In contrast, the eutherian mother is primed during pregnancy to display sophisticated maternal nurturing from birth, raising the intriguing possibility that imprinting of *Phlda2* contributed to the evolution of maternal care in mammals. *Phlda2*, *pleckstrin homology-like domain family A member 2*.

In summary, our data provide further support for conflict related to pregnancy in mammals [81] and the prediction that placental hormones (or genes that regulate placental hormones) might be imprinted [82]. This is also a very rare, possibly unique, example of increased maternal care prenatally programmed by a single gene modification in the offspring. *Phlda2* acquired an imprinted status during the transition from a marsupial to eutherian mode of reproduction [83], raising the intriguing possibility that imprinting of *Phlda2* contributed to increased maternal care during the evolution of mammals (Fig 5). As a final point, this study may also have important implications for human health. Elevated placental *PHLDA2* is a common findings in foetal growth restriction [84], which is associated with mood disorders in human pregnancies [85]. It will be important to know whether human mothers carrying infants with elevated placental *PHLD*A2 also show deficits in maternal behaviour.

## Materials and methods

### Ethics statement

Animal studies and breeding were approved by the University of Cardiff’s ethical committee, performed under a United Kingdom Home Office project license (RMJ; PPL 3003134), and abide by ARRIVE guidelines. At the end of the study, animals were euthanised as required by the UK Home Office.

### Mouse strains and genotyping

Mice were housed in a conventional unit on a 12-hour light–dark cycle with lights coming on at 07:00 hours, with a temperature range of 21 °C +/− 2 °C, with free access to tap water and standard chow. The *Phlda2*-targeted allele [52] and the *Phlda2* BAC transgenic line [53] were maintained (>12 generations) and studied on the 129S2/SvHsd (129) strain background, with control embryos and recipient females generated from a concurrently maintained, fully WT 129 colony. Embryos and pups were genotyped as previously described [52, 53].

### Recipient transfer

To generate experimental dams, 5–7-week-old virgin female mice of the appropriate genotype (*Phlda2*^+/+^, *Phlda2*^−/−^, or *Phlda2*^+/+BACx1^) were superovulated by 5 units (100 μl) intraperitoneal pregnant mare serum followed by 5 units (100 μl) intraperitoneal human chorionic gonadotropin 44–48 hours later, and mated with either WT or *Phlda2*^+/+BACx1^ stud males. Embryos were flushed from oviducts in M2 media (Sigma) 2 days after a discernible plug on E1.5 and incubated at 37 °C under 5% CO_2_ for 1–2 hours in KSOM (Specialty Media) before 14–16 embryos were transferred via bilateral oviduct transfer into E0.5 pseudopregnant WT 129 females aged 5–7 weeks. *Phlda2*^+/+BACx1^ animals carry a single copy of a nonimprinted 85-kb transgene spanning the genomic *Phlda2* locus [54]. Homozygosity was found to be unviable, and 2 heterozygous animals were mated to generate embryos with only litters with >60% transgenic pups used in the downstream analysis. Pregnant females were either killed at E16.5 or taken to term for the behavioural analysis. Day of delivery (D0) and litter size were recorded. Dams with litter sizes between 6 and 12 live pups were used for analysis, and there were no significant differences in litter size within any experiment (Data in S2 and S3 Tables). All experimental samples were generated by recipient transfer.

### Microarray

Dams were weighed and euthanised at E16.5 by cervical dislocation; whole brains were rapidly removed, followed by fine dissection of the hypothalamus and hippocampus, which were rapidly frozen on dry ice. RNA was prepared by homogenisation in RNA-Bee (Ams Biotechnology) following manufacturer’s instructions, and RNA concentration was evaluated using a NanoDrop Spectrophotometer, with RNA concentration adjusted to approximately 1 μg/μl using 10 mM Tris (pH 8). Gene expression analysis was performed using the Affymetrix GeneChip Mouse Gene 2.0 ST Array. Microarray data analysis was performed using the R programming language. Linear Model for Microarray data Analysis (LIMMA) was used to identify differentially expressed (DE) genes between all pairwise comparisons across the sample groups to generate heat maps of changes in gene expression levels at a *p* value <0.05. Pathway analysis was performed using Database for Annotation Visualization and Integrated Discovery (DAVID) and iPathwayGuide [86–88]. Impact analysis uses two types of evidence to define the most greatly affected pathways affected in a system: (i) the overrepresentation of DE genes in a given pathway and (ii) the perturbation of that pathway, computed by propagating the measured expression changes across the pathway topology. The first probability, pORA, expresses the probability of observing the number of DE genes in a given pathway that is greater than or equal to the one observed by random chance. The second probability, pAcc, was calculated based on the amount of total accumulation measured in each pathway. A perturbation factor was computed for each gene on the pathway. The two types of evidence, pORA and pAcc, were combined into one final pathway score by calculating a *p* value using Fisher’s method. This *p* value was then corrected for multiple comparisons using false discovery rate (FDR) or Bonferroni corrections.

### EPM

The EPM test was carried out on P2 when dams were in their home cage between 8:00 AM and 10:00 AM hr to evaluate anxiety levels. Each new dam was given 10–15 minutes to habituate to the test room before embarking on the test away from her pups. The EPM consisted of 2 opposite open arms and 2 opposite closed arms (19 cm × 8 cm × 15 cm; length × width × height) elevated 1 metre above ground. The middle section that allows the animal to transit from arm to arm consisted of a square with dimensions of 12 × 12 cm. Testing was carried out under low light conditions (230 lux on open sections). Each mouse was placed in the centre of the maze, and the amount of time spent in each arm was recorded automatically by EthoVision XT 8.0 video tracking software (Noldus Information Technology, Netherlands) over 5 minutes. Frequency and duration of rearing, stretch-attend, and head-dips over the end or side of the open arms were recorded.

### Pup retrieval

Pup retrieval and nest building took place on P3 between 8:00 AM and 10:00 AM after a 24-hour habitation period in the Phenotyper cage (Noldus, Netherlands). Cages were equipped with cameras in the roof for video recording, and activity was tracked using Ethovision XT software (Noldus Information Technology, Netherlands). The dam was removed from her cage, and her pups were removed from the nest. Pups were placed at the opposite end of the cage. The female was returned to her cage in the opposite corner to the pups and nest, and the latency to sniff and then retrieve the first pup was recorded. Video recording was continued for the following 23 hours before the nest building task, and videos were analysed by 2 researchers blind to the experimental groups.

### Nest building

The quality of the nest building was scored on P4 between 8:00 AM and 10:00 AM. The Phenotyper recording was paused 23 hours into the recording. Dams, pups, and nests were removed to the original home cage. A cardboard tube (International Product Supplies, UK) and 30 x 30 cm strips of tissue paper were placed in the Phenotyper cage, pups were placed next to the new bedding material, dams were returned to the cage, and recording resumed for 1 hour. Nest quality and presence of pups in the nest were scored from videos, using a simple scale of no nest, nest built but no pups, or nest built and pups inside the nest, by 2 researchers blind to the genotype of the pups. Distance moved during this trial and visits to virtual ‘zones’ were calculated automatically using Ethovision XT software. Behaviours scored were total, crouched, arched, and passive nursing, grooming of pups, contacts with pups, self-grooming, and visits to food hopper or water zone.

### WT pup fostering

WT(1x) and WT(0x) dams were generated by recipient transfer by the same protocol used to generate the original groups. Dams were checked every 6–8 hours from E19.5 for the presence of pups. When newly born pups were found at the same time in both models, WT pups were removed from their dams, and 6 WT pups were either fostered to a WT(0x) dam or to a different WT(1x) dam (control). All litters were then left undisturbed in Phenotypers until testing from P3.

### USV recording

USV was performed on fully WT pups and fully *Phlda2*^−/−^ pups on P2 in a separate cohort generated by natural mating. The dam’s litter was removed from the home cage and placed in a separate home cage. USVs made by the pups were recorded for 180 seconds using Avisoft-UltraSoundGate 116Hb (Avisoft Bioacoustics e.K., Germany).

### Statistical analysis

The experimental data for the all the behavioural tests were analysed using SPSS Version 23 (SPSS, United States of America) unless otherwise stated. All behavioural data were presented as mean values with the standard error of the mean (± SEM) also displayed. Each behavioural test was analysed separately according to genotype, followed by any appropriate comparisons between genotypes or across genotypes. This was done using separate ANOVAs for various between-subject factors of genotype and within-subject factors.

## Supporting information

S1 Table Some examples of rodent models reporting altered maternal behavioural phenotypes. Examples of enhanced or induced maternal behaviour highlighted in bold text.
(DOCX)

S2 Table Data from behavioural assessment of wild-type dams exposed to different doses of *Phlda2* in their offspring. *Phlda2*, *pleckstrin homology-like domain family A member 2*.
(DOCX)

S3 Table Data from behavioural assessment of WT dams exposed to different doses of *Phlda2* prenatally and WT pups postpartum. *Phlda2*, *pleckstrin homology-like domain family A member 2*; WT, wild-type.
(DOCX)

S1 Fig Gene expression levels in mature mouse placenta. Mouse ENCODE transcriptome data. In red, genes with a lower RPKM value than 1 (genes that are not expressed in placenta); in black, genes with a higher RPKM value than 1. (A) Graph representation of gene expression levels of the different pathways represented plus gene expression levels of placental genes. (B) Steroid hormones. (C) Serotonin. (D) Dopamine, noradrenaline, adrenaline. (E) Oxytocin (labelled ‘Oxy’). (F) Arginine vasopressin (labelled ‘Arg/Vas’). *Altered in *Phlda2* mutant placenta. (G) Expression levels in mature mouse placenta. Mouse ENCODE transcriptome data. Count: approximate total reads mapped to gene transcript features. In red genes with a lower RPKM value than 1 (genes that are not expressed in placenta), in black genes with a higher RPKM value than 1. *Phlda2*, *pleckstrin homology-like domain family A member 2*; RPKM, reads per kilobase per million reads placed.
(TIFF)

S2 Fig Litter size, foetal, and pup weights. (A) Significant difference in foetal weight at E16.5 across the three models (F2, 254 = 4.7, *p* = 0.045). (B) No difference in litter size across three models (F2, 36 = 0.83, *p* = 0.45). (C) Adequate catch-up growth of both *Phlda2*^−/+^ and *Phlda2*^+/+BACx1^ pups by P7. Numerical data can be found at https://osf.io/543jg/ “RAW NUMERICAL DATA.xlsx”, Sheets labelled Litter_Size_Weight. E, embryonic day; P, postnatal day.
(TIFF)

S3 Fig Signalling pathways, biological processes, and molecular functions altered in maternal hypothalamus and hippocampus with *P* values. Raw data can be found using accession number: GSE115276.
(TIF)

S4 Fig USVs at P2 and P4 from naturally mated fully wild-type and *Phlda2*^*−/+*^ (KO) litters. Wild-type pups were generated by mating wild-type, virgin females, aged 5–7 weeks, with wild-type, experienced studs (*n* = 4). *Phlda2*^−/+^ pups were generated by mating homozygous *Phlda2*^−/−^ virgin females aged 5–7 weeks with wild-type, experienced studs (*n* = 4). Pup USVs were recorded using Avisoft-UltraSoundGate 116Hb (Avisoft Bioacoustics e.K., Germany) over 180 seconds on P2 and P4. Numerical data can be found at https://osf.io/543jg/ “RAW NUMERICAL DATA.xlsx”, Sheets labelled USV. KO, knockout; P, postnatal day; USV, ultrasonic vocalisation.
(TIFF)

S1 ARRIVE Checklist (PDF)

## Abbreviations

129S2/SvHsd: 129
BAC: bacterial artificial chromosome
DAVID: Database for Annotation Visualization and Integrated Discovery
DE: differentially expressed
E: embryonic day
EPM: elevated plus maze
FDR: false discovery rate
GO: gene ontology
GPCR: G protein–coupled receptor
KO: knockout
P: postnatal day
*Peg1*: *paternally expressed gene 1*
*Peg3*: *paternally expressed gene 3*
PH: pleckstrin homology
*PHLDA2*: *pleckstrin homology-like domain family A member 2*
USV: ultrasonic vocalisation
WT(0x): WT dams carrying and caring for all *Phlda2*^−/+^ embryos (maternal inheritance of targeted allele, *Phlda2* loss of function)
WT(0x)^WT^: WT dams carrying all *Phlda2*^−/+^ embryos and caring for all WT pups
WT(1x): WT dams carrying and caring for all WT embryos
WT(1x)^WT^: WT dams carrying all WT embryos and caring for all WT pups from a different WT litter
WT(2x): WT dams carrying >60% *Phlda2*^+/+BACx1^ embryos generated by crossing *Phlda2*^+/+BACx1^ dams and *Phlda2*^+/+BACx1^ studs
WT: wild-type
