# Supplementary material for: Maternal care boosted by paternal imprinting in mammals
Source: PLoS Biol. 2018 Jul 31;16(7):e2006599. doi: 10.1371/journal.pbio.2006599 (PMC6067684; doi:10.1371/journal.pbio.2006599)
Supplement: S1 Table — Examples of enhanced or induced maternal behaviour highlighted in bold text. (DOCX) [file pbio.2006599.s001.docx]

| Genetic alteration in dam  (KO unless otherwise stated) | Maternal behavioural alteration | Species | Reference |
| --- | --- | --- | --- |
| Adenylyl cyclase 3 | pup retrieval deficit; nest building deficit; aggression deficit | Mice | Wang Z, Storm DR. Maternal behavior is impaired in female mice lacking type 3 adenylyl cyclase. Neuropsychopharmacology. 2011 Mar;36(4):772-81. |
| (S)-alpha-amino-3-hydroxy-5-methyl-isoxazolepropionic acid (AMPA) receptors | pup retrieval deficit | Mice | Shimshek DR, Bus T, Grinevich V, Single FN, Mack V, Sprengel R, Spergel DJ, Seeburg PH. Impaired reproductive behavior by lack of GluR-B containing AMPA receptors but not of NMDA receptors in hypothalamic and septal neurons. Mol Endocrinol. 2006 Jan;20(1):219-31. |
| Alpha 1 microglobulin/bikunin | nurturing deficit | Mice | Zhuo L, Yoneda M, Zhao M, Yingsung W, Yoshida N, Kitagawa Y, Kawamura K, Suzuki T, Kimata K. Defect in SHAP-hyaluronan complex causes severe female infertility. A study by inactivation of the bikunin gene in mice. J Biol Chem. 2001 Mar 16;276(11):7693-6 |
| CD38  (oxytocin release) | pup retrieval deficit; nurturing deficit | Mice | Jin D, Liu HX, Hirai H, Torashima T, Nagai T, Lopatina O, Shnayder NA, Yamada K, Noda M, Seike T, Fujita K, Takasawa S, Yokoyama S, Koizumi K, Shiraishi Y, Tanaka S, Hashii M, Yoshihara T, Higashida K, Islam MS, Yamada N, Hayashi K, Noguchi N, Kato I, Okamoto H, Matsushima A, Salmina A, Munesue T, Shimizu N, Mochida S, Asano M, Higashida H. CD38 is critical for social behaviour by regulating oxytocin secretion. Nature. 2007 Mar 1;446(7131):41-5. |
| Circling  (mutation) | nurturing deficit | Mice | Lee JW, Lee EJ, Hong SH, Chung WH, Lee HT, Lee TW, Lee JR, Kim HT, Suh JG, Kim TY, Ryoo ZY. Circling mouse: possible animal model for deafness.Comp Med. 2001 Dec;51(6):550-4. |
| Cortocotropin-releasing hormone receptor 2 | decreased maternal aggression | Mice | Gammie SC, Hasen NS, Stevenson SA, Bale TL, D'Anna KL. Elevated stress sensitivity in corticotropin-releasing factor receptor 2 deficient mice decreases maternal, but not intermale aggression. Behav Brain Res. 2005 May 7;160(1):169-77. |
| Cyclic AMP response element-binding protein | nurturing deficit | Mice | Jin SH, Blendy JA, Thomas SA. Cyclic AMP response element-binding protein is required for normal maternal nurturing behavior. Neuroscience. 2005;133(3):647-55. |
| Cyclin D1 | nurturing deficit (lack of milk) | Mice | Fantl V, Stamp G, Andrews A, Rosewell I, Dickson C. Mice lacking cyclin D1 are small and show defects in eye and mammary gland development. Genes Dev. 1995 Oct 1;9(19):2364-72. |
| Dopamine beta hydroxylase | pup retrieval deficit; nurturing deficit; deficit in placentophagia | Mice | Thomas SA, Palmiter RD. Impaired maternal behavior in mice lacking norepinephrine and epinephrine.Cell. 1997 Nov 28;91(5):583-92. |
| Dopamine transporter | pup retrieval deficit | Mice | Spielewoy C, Roubert C, Hamon M, Nosten-Bertrand M, Betancur C, Giros B.Behavioural disturbances associated with hyperdopaminergia in dopamine-transporter knockout mice.Behav Pharmacol. 2000 Jun;11(3-4):279-90. |
| Ephrin-A5 | nest building deficit; pup retrieval deficit; decreased anxiety | Mice | Sheleg M, Yu Q, Go C, Wagner GC, Kusnecov AW, Zhou R. Decreased maternal behavior and anxiety in ephrin-A5-/- mice. Genes Brain Behav. 2017 Feb;16(2):271-284. |
| Estrogen receptor 1 (alpha) | pup retrieval deficit; nurturing deficit; infanticide | Mice | Ogawa S, et al. (1998) Roles of estrogen receptor-alpha gene expression in reproduction-related behaviors in female mice. Endocrinology 139:5070–5081. |
| Estrogen receptor 1 (alpha)  (acute MPOA; siRNA) | nurturing deficit; pup retrieval deficit | Mice | Ribeiro AC, Musatov S, Shteyler A, Simanduyev S, Arrieta-Cruz I, Ogawa S, Pfaff DW. siRNA silencing of estrogen receptor-α expression specifically in medial preoptic area neurons abolishes maternal care in female mice. Proc Natl Acad Sci U S A. 2012 Oct 2;109(40):16324-9. |
| Estrogen receptor 1 (alpha)  (optogenetic inactivation +ve cells in MPOA; postpartum) | Failure to retrieve; unchanged pup grooming, sniffing, and crouching | Mice |  |
| Fibroblast Growth Factor Receptors  (dominant negative) | pup retrieval deficit; pups scattered | Mice | Brooks LR, Le CD, Chung WC, Tsai PS. Maternal behavior in transgenic mice with reduced fibroblast growth factor receptor function in gonadotropin-releasing hormone neurons. Behav Brain Funct. 2012 Sep 5;8:47 |
| Forkhead box B1 | nurturing deficit | Mice | Wehr R, Mansouri A, de Maeyer T, Gruss P.Fkh5-deficient mice show dysgenesis in the caudal midbrain and hypothalamic mammillary body.Fkh5-deficient mice show dysgenesis in the caudal midbrain and hypothalamic mammillary body. |
| FosB | pup retrieval deficit; nurturing deficit; deficit in placentophagia | Mice | Brown JR, Ye H, Bronson RT, Dikkes P, Greenberg ME.A defect in nurturing in mice lacking the immediate early gene fosB.Cell. 1996 Jul 26;86(2):297-309. |
| Free fatty acid receptor 1 | pup retrieval deficit; infanticide; decreased anxiety; | Mice | Aizawa F, Ogaki Y, Kyoya N, Nishinaka T, Nakamoto K, Kurihara T, Hirasawa A, Miyata A, Tokuyama S.The Deletion of GPR40/FFAR1 Signaling Damages Maternal Care and Emotional Function in Female Mice. Biol Pharm Bull. 2017;40(8):1255-1259 |
| Fyn tyrosine kinase and hexanal | pup retrieval deficit; nest building deficit | Mice | Hamaguchi-Hamada K, Sanbo C, Hamada S, Yagi T.Exposure to hexanal odor influences maternal behavior and induces neonatal death in Fyn tyrosine kinase-deficient mice.Neurosci Res. 2004 Mar;48(3):259-67. |
| GABA A receptor  (gain-of-function) | nurturing deficit | Mice | Homanics GE, Elsen FP, Ying SW, Jenkins A, Ferguson C, Sloat B, Yuditskaya S, Goldstein PA, Kralic JE, Morrow AL, Harrison NL.A gain-of-function mutation in the GABA receptor produces synaptic and behavioral abnormalities in the mouse. Genes Brain Behav. 2005 Feb;4(1):10-9. |
| GABA A receptor, subunit beta 3 | nurturing deficit | Mice | Homanics GE, DeLorey TM, Firestone LL, Quinlan JJ, Handforth A, Harrison NL, Krasowski MD, Rick CE, Korpi ER, Mäkelä R, Brilliant MH, Hagiwara N, Ferguson C, Snyder K, Olsen RW. Mice devoid of gamma-aminobutyrate type A receptor beta3 subunit have epilepsy, cleft palate, and hypersensitive behavior.Proc Natl Acad Sci U S A. 1997 Apr 15;94(8):4143-8. |
| Galpha(q) and Galpha(11)  (alpha-subunits; forebrain) | no pup retrieval, nest building or nurturing | Mice | Wettschureck N, Moers A, Hamalainen T, Lemberger T, Schütz G, Offermanns S. Heterotrimeric G proteins of the Gq/11 family are crucial for the induction of maternal behavior in mice. Mol Cell Biol. 2004 Sep;24(18):8048-54. |
| GABA A receptor, subunit delta | Postpartum depressive behaviour; decreased aggression; increased anxiety; poor nest building; canabilism; | Mice | Maguire J, Mody I. 2008. GABA(A)R plasticity during pregnancy: relevance to postpartum depression. Neuron 59: 207–13. |
| Hairless | nurturing deficit | Mice | Grew FAE and Mirskaia L..The character “hairless” in the mouse. Journal of Genetics 1931 Nov;25(1):17–24 |
| Heterochromatin protein 1 binding protein 3 | nest building deficit; pup retrieval deficit; decreased anxiety | Mice | Garfinkel BP, Arad S, Neuner SM, Netser S, Wagner S, Kaczorowski CC, Rosen CJ, Gal M, Soreq H, Orly J. HP1BP3 expression determines maternal behavior and offspring survival. Genes Brain Behav. 2016 Sep;15(7):678-88 |
| Melanin concentrating hormone receptor 1 | nurturing deficit; pup retrieval deficit; decreased aggression | Mice | Alachkar A, Alhassen L, Wang Z, Wang L, Onouye K, Sanathara N, Civelli O. Inactivation of the melanin concentrating hormone system impairs maternal behavior. Eur Neuropsychopharmacol. 2016 Nov;26(11):1826-1835. |
| Methyl CpG-binding protein 2 (heterozygous) | pup retrieval deficit | Mice | Krishnan K, Lau BY, Ewall G, Huang ZJ, Shea SD. MECP2 regulates cortical plasticity underlying a learned behaviour in adult female mice. Nat Commun. 2017 Jan 18;8:14077. doi: 10.1038/ncomms14077. |
| Microphthalmia-associated transcription factor | nurturing deficit | Mice | Hansdottir AG, Pálsdóttir K, Favor J, Neuhäuser-Klaus A, Fuchs H, de Angelis MH, Steingrímsson E. The novel mouse microphthalmia mutations Mitfmi-enu5 and Mitfmi-bcc2 produce dominant negative Mitf proteins. Genomics. 2004 May;83(5):932-5. |
| Microtubule-associated protein 6 | nurturing deficit; pup retrieval deficit; increased anxiety | Mice | Andrieux A, Salin PA, Vernet M, Kujala P, Baratier J, Gory-Fauré S, Bosc C, Pointu H, Proietto D, Schweitzer A, Denarier E, Klumperman J, Job D. The suppression of brain cold-stable microtubules in mice induces synaptic defects associated with neuroleptic-sensitive behavioral disorders. Genes Dev. 2002 Sep 15;16(18):2350-64. |
| Neuromedin B receptor after restraint stress | nurturing deficit | Mice | Yamada K, Santo-Yamada Y, Wada K. Restraint stress impaired maternal behavior in female mice lacking the neuromedin B receptor (NMB-R) gene.Neurosci Lett. 2002 Sep 20;330(2):163-6. |
| Neuronal nitric oxide synthase | decreased aggression | Mice | Gammie SC, Nelson RJ. Maternal aggression is reduced in neuronal nitric oxide synthase-deficient mice. J Neurosci. 1999 Sep 15;19(18):8027-35. |
| Neuronal PAS domain protein 1 & 3 | nurturing deficit | Mice | Erbel-Sieler C, Dudley C, Zhou Y, Wu X, Estill SJ, Han T, Diaz-Arrastia R, Brunskill EW, Potter SS, McKnight SL. Behavioral and regulatory abnormalities in mice deficient in the NPAS1 and NPAS3 transcription factors. Proc Natl Acad Sci U S A. 2004 Sep 14;101(37):13648-53. |
| NMDA receptor  (mutation) | nurturing deficit; pup retrieval deficit; increased aggression | Mice | Single FN, Rozov A, Burnashev N, Zimmermann F, Hanley DF, Forrest D, Curran T, Jensen V, Hvalby O, Sprengel R, Seeburg PH. Dysfunctions in mice by NMDA receptor point mutations NR1(N598Q) and NR1(N598R).J Neurosci. 2000 Apr 1;20(7):2558-66. |
| Nuclear receptor subfamily 2, group E, member 1 | nurturing deficit; increased aggression; decreased anxiety | Mice | Young KA, Berry ML, Mahaffey CL, Saionz JR, Hawes NL, Chang B, Zheng QY, Smith RS, Bronson RT, Nelson RJ, Simpson EM.Fierce: a new mouse deletion of Nr2e1; violent behaviour and ocular abnormalities are background-dependent.Behav Brain Res. 2002 May 14;132(2):145-58 |
| Orthodenticle homolog 3 | nurturing deficit | Mice | Ohtoshi A, Behringer RR.Neonatal lethality, dwarfism, and abnormal brain development in Dmbx1 mutant mice.Mol Cell Biol. 2004 Sep;24(17):7548-58. |
| Oxytocin  (postpartum) | normal maternal behaviour (24 hr); disrupted milk release; infanticide | Mice | Nishimori K, Young LJ, Guo Q, Wang Z, Insel TR, Matzuk MM.Oxytocin is required for nursing but is not essential for parturition or reproductive behavior.Proc Natl Acad Sci U S A. 1996 Oct 15;93(21):11699-704. |
| Oxytocin  (virgin) | modest deficiencies in pup retrieval | Mice | Takayanagi Y, Yoshida M, Bielsky IF, Ross HE, Kawamata M, Onaka T, Yanagisawa T, Kimura T, Matzuk MM, Young LJ, Nishimori K. Pervasive social deficits, but normal parturition, in oxytocin receptor-deficient mice. 2005 Proc Natl Acad Sci USA 102:16096–16101. |
| Oxytocin  (virgin) | impaired maternal behaviour in seminatural environment; infanticide | Mice | Ragnauth AK, Devidze N, Moy V, Finley K, Goodwillie A, Kow LM, Muglia LJ, Pfaff DW. 2005. Female oxytocin gene-knockout mice, in a semi-natural environment, display  exaggerated aggressive behavior. Genes Brain Behav 4:229–239. |
| Oxytocin  (postpartum; foster pups) | delayed retrieval; reduced licking (only in novel environment) | Mice | Pedersen CA, Vadlamudi SV, Boccia ML, Amico JA.Maternal behavior deficits in nulliparous oxytocin knockout mice.Genes Brain Behav. 2006 Apr;5(3):274-81. |
| Oxytocin receptor  (forebrain) | normal maternal behaviour; infanticide | Mice | Macbeth AH, Stepp JE, Lee HJ, Young WS 3RD, Caldwell HK. Normal maternal behavior, but increased pup mortality, in conditional oxytocin receptor knockout females. 2010. Behav Neurosci 124:677–685. |
| Oxytocin receptor  (postpartum; foster pups) | delayed retrieval; delayed in crouching; decreased duration crouching | Mice | Takayanagi Y, Yoshida M, Bielsky IF, Ross HE, Kawamata M, Onaka T, Yanagisawa T, Kimura T, Matzuk MM, Young LJ, Nishimori K. Pervasive social deficits, but normal parturition, in oxytocin receptor-deficient mice. 2005 Proc Natl Acad Sci USA 102:16096–16101. |
| Oxytocin receptor  (postpartum; foster pups; thelectomized control) | normal maternal behaviours; pup abandonment | Mice | Rich ME, Decardenas EJ, Lee HJ, Caldwell HK. Impairments in the initiation of maternal behavior in oxytocin receptor knockout mice. 2014 PLoS One 9:e98839. |
| Paternally expressed imprinted gene 1 | pup retrieval deficit; nest building deficit; nurturing deficit | Mice | Lefebvre L, Viville S, Barton SC, Ishino F, Keverne EB, Surani MA. Abnormal maternal behaviour and growth retardation associated with loss of the imprinted gene Mest. Nat Genet. 1998 Oct;20(2):163-9. |
| Paternally expressed imprinted gene 3  (129 strain) | pup retrieval deficit; nest building deficit; nurturing deficit | Mice | Li L, Keverne EB, Aparicio SA, Ishino F, Barton SC, Surani MA.Regulation of maternal behavior and offspring growth by paternally expressed Peg3.Science. 1999 Apr 9;284(5412):330-3. |
| Paternally expressed imprinted gene 3  (BL6 strain) | no deficit | Mice | Denizot AL, Besson V, Correra RM, Mazzola A, Lopes I, Courbard JR, Marazzi G, Sassoon DA. A Novel Mutant Allele of Pw1/Peg3 Does Not Affect Maternal Behavior or Nursing Behavior. PLoS Genet. 2016 May 17;12(5):e1006053 |
| Paternally expressed imprinted gene 3  (KO in offspring only) | delay to sniff and retrieve; increased anxiety; no nurturing deficit | Mice | McNamara GI, Creeth HDJ, Harrison DJ, Tansey KE, Andrews RM, Isles AR, John RM. Loss of offspring Peg3 reduces neonatal ultrasonic vocalisations and increases maternal anxiety in wild type mothers. Hum Mol Genet. 2018 Feb 1;27(3):440-450 |
| Pet1 ETS transcription factor | pup retrieval deficit; nest building deficit; nurturing deficit | Mice | Lerch-Haner JK, Frierson D, Crawford LK, Beck SG, Deneris ES. Nat Neurosci. 2008; 11:1001–1003. |
| Pituitary adenylate cyclase-activating polypeptide | decreased crouching | Mice | Shintani N, Mori W, Hashimoto H, Imai M, Tanaka K, Tomimoto S, Hirose M, Kawaguchi C, Baba A.Defects in reproductive functions in PACAP-deficient female mice.Regul Pept. 2002 Nov 15;109(1-3):45-8. |
| Prolactin | no deficit in maternal care | Mice | Horseman ND, Zhao W, Montecino-Rodriguez E, Tanaka M, Nakashima K, Engle SJ, Smith F, Markoff E, Dorshkind K. Defective mammopoiesis, but normal hematopoiesis, in mice with a targeted disruption of the prolactin gene. The EMBO Journal. 1997; 16:6926–6935 |
| Prolactin receptor | nurturing deficit; pup retrieval deficit | Mice | Bole-Feysot C, Goffin V, Edery M, Binart N, Kelly PA.Prolactin (PRL) and its receptor: actions, signal transduction pathways and phenotypes observed in PRL receptor knockout mice.Endocr Rev. 1998 Jun;19(3):225-68. |
| Prolactin receptor  (heterozygous/virgin) | deficit in pup retrieval and crouching (some but not all) | Mice | Lucas BK, Ormandy CJ, Binart N, Bridges RS, Kelly PA.Null mutation of the prolactin receptor gene produces a defect in maternal behavior.Endocrinology. 1998 Oct;139(10):4102-7. |
| Prolactin receptor  (glutamatergic neurons) | no effect on pup retrieval in home cage or novel cage |  | Brown RSE, Aoki M, Ladyman SR, Phillipps HR, Wyatt A, Boehm U, Grattan, DR. Prolactin action in the medial preoptic area is necessary for postpartum maternal nursing behavior. PNAS 2017 October, 114 (40) 10779-10784. |
| Prolactin receptor  (GABAnergic neurons) | some abandoned litters; no effect on pup retrieval in home or novel cage; delay to crouch and exhibit full maternal behaviour in novel cage | Mice |  |
| Prolactin receptor  (MPOA; acute) | initiated retrieval, cleaning, grooming and placentophagia; failed to establish full maternal care (crouching and arched back nursing; death of pups | Mice |  |
| Serotonin 1b receptor | decreased time in nest; hyperactive; decreased anxiety | Mice | Brunner D, Buhot MC, Hen R, Hofer M.Anxiety, motor activation, and maternal-infant interactions in 5HT1B knockout mice.Behav Neurosci. 1999 Jun;113(3):587-601. |
| Testicular orphan nuclear receptor 4 | no nests; no pup collection; no crouching or nursing | Mice | Collins LL, Lee YF, Heinlein CA, Liu NC, Chen YT, Shyr CR, Meshul CK, Uno H, Platt KA, Chang C.Growth retardation and abnormal maternal behavior in mice lacking testicular orphan nuclear receptor 4.Proc Natl Acad Sci U S A. 2004 Oct 19;101(42):15058-63. |
| Transient receptor potential 2 channel | decreased pup interaction; decreased aggression | Mice | Leypold, B. G., Yu, C. R., Leinders-Zufall, T., Kim, M. M., Zufall, F., and Axel, R. (2002). Altered sexual and social behaviors in trp2 mutant mice. Proc. Natl. Acad. Sci. USA99, 6376-6381 |
| Tryptophan hydroxylase 2 | decreased survival; nurturing deficit; nest building deficit; pup retrieval deficit | Mice | Angoa-Pérez M, Kane MJ, Sykes CE, Perrine SA, Church MW, Kuhn DM. Brain serotonin determines maternal behavior and offspring survival.Genes Brain Behav. 2014 Sep;13(7):579-91 |
| tyrosine hydroxylase  (Striatum; popamine transporter nuerons) | delayed retrieval; impaired in licking/grooming and nursing; locomotor activity unaffected | Mice | Henschen CW, Palmiter RD, Darvas M. Restoration of Dopamine Signaling to the Dorsal Striatum Is Sufficient for Aspects of Active Maternal Behavior in Female Mice. Endocrinology. 2013 Nov;154(11):4316-27 |
| Type 3 deiodinase | no pup-retrieval; increased aggression toward newborns | Mice | Stohn JP, Martinez ME, Zafer M, López-Espíndola D, Keyes LM, Hernandez A. Increased aggression and lack of maternal behavior in Dio3-deficient mice are associated with abnormalities in oxytocin and vasopressin systems. Genes Brain Behav. 2018 Jan;17(1):23-35. |
| Ubiquitin-Specific Peptidase 46 | nurturing deficit (nursing/grooming); reduced pup survival | Mice | Umemura S, Imai S, Mimura A, Fujiwara M, Ebihara S (2015) Impaired Maternal Behavior in Usp46 Mutant Mice: A Model for Trans-Generational Transmission of Maternal Care. PLoS ONE 10(8): e0136016. |
| Urocortin (Ucn) 1 and Ucn 3 | decreased aggression | Mice | D'Anna KL, Stevenson SA, Gammie SC. Urocortin 1 and 3 impair maternal defense behavior in mice.Behav Neurosci. 2005 Aug;119(4):1061-71 |
| Vomeronasal 1 receptors  (subset) | decreased aggression | Mice | Del Punta K, Leinders-Zufall T, Rodriguez I, Jukam D, Wysocki CJ, Ogawa S, Zufall F, Mombaerts P.Deficient pheromone responses in mice lacking a cluster of vomeronasal receptor genes.Nature. 2002 Sep 5;419(6902):70-4. |
| Non-genetic alteration to dam | Maternal behavioural alteration | Species | Reference |
| 2,4-dichloro+A57:D65 phenoxyacetic acid  (herbicide in food; postpartum) | reduced retrieval; reduced crouching and licking | Rat | Stürtz N, Deis RP, Jahn GA, Duffard R, Evangelista de Duffard AM. Effect of 2,4-dichlorophenoxyacetic acid on rat maternal behavior.Toxicology. 2008 May 21;247(2-3):73-9. |
| Bisphenol A  (oral; late pregnancy) | nursing deficit; less time on the nest | Mice | Palanza PL, Howdeshell KL, Parmigiani S, vom Saal FS. Exposure to a low dose of bisphenol A during fetal life or in adulthood alters maternal behavior in mice. Environ Health Perspect. 2002;110 (Suppl 3):415–422 |
| Bisphenol S  (pregnancy and lactation) | poor cleaning of the pups; more time on the nest; smaller nest; delayed pup retrieval; infanticide | Mice | Catanese MC, Vandenberg LN. Bisphenol S (BPS) Alters Maternal Behavior and Brain in Mice Exposed During Pregnancy/Lactation and Their Daughters. Endocrinology. 2017 Mar 1;158(3):516-530. |
| Cocaine  (IP; postpartum) | nurturing deficit (retrieving, grouping, and crouching over six pups) | Rat | Kinsley CH, Turco D, Bauer A, Beverly M, Wellman J, Graham AL.Cocaine alters the onset and maintenance of maternal behavior in lactating rats.Pharmacol Biochem Behav. 1994 Apr;47(4):857-64. |
| Corticosterone  (subcutaneous.; pregnancy +/- lactation) | reduced nursing; increased time off nest | Rat | Brummelte, S. & Galea, L. A. Chronic corticosterone during pregnancy and postpartum affects maternal care, cell proliferation and depressive-like behavior in the dam. Horm. Behav. 58, 769–779 (2010). |
| Diet/high fat  (pregnancy and lactation) | **increased duration nursing (arched) in dark phase** | Rat | Purcell RH, Sun B, Pass LL, Power ML, Moran TH, Tamashiro KL. Maternal stress and high-fat diet effect on maternal behavior, milk composition, and pup ingestive behavior. Physiol Behav. 2011 Sep 1; 104(3): 474–479. |
| Diet/high fat  (pregnancy and lactation) | **decreased anxiety; increased arched back nursing, licking–grooming and passive nursing (dark phase)** | Rat | Rincel M, Lépinay AL, Delage P, Fioramonti J, Théodorou VS, Layé S, Darnaudéry M. Maternal high-fat diet prevents developmental programming by early-life stress. Transl Psychiatry. 2016 Nov; 6(11): e966. |
| Diet/high fat and protein  (pre-pregnancy through lactation) | **increased nursing and grooming** | Rat | Bertino M. Effects of high fat, protein supplemented diets on maternal behavior in rats.Physiol Behav. 1982 Dec;29(6):999-1005. |
| Dopamine receptor antagonist  (cis-flupenthixol; nucleus accumbens; postpartum) | delayed retrieval; **increased duration of licking and nursing** | Rat | Keer SE, Stern JM. Dopamine receptor blockade in the nucleus accumbens inhibits maternal retrieval and licking, but enhances nursing behavior in lactating rats. 1999 Physiol Behav 67: 659–69. |
| Dopamine D1 receptor antagonist  (SCH 23390; nucleus accumbens; pregnant) | reduced grooming; delayed retrieval | Rat | Byrnes, EM, Rigero BA, Bridges, RS. Dopamine antagonists during parturition disrupt maternal care and the retention of maternal behavior in rats. Pharmacol Biochem Behav. 2002 Nov;73(4):869-75. |
| Dopamine D1 receptor antagonist  (SCH 23390; nucleus accumbens; postpartum) | disrupted retrieving | Rat | Numan M, Numan MJ, Pliakou N, Stolzenberg DS, Mullins OJ, Murphy JM, Smith CD.The effects of D1 or D2 dopamine receptor antagonism in the medial preoptic area, ventral pallidum, or nucleus accumbens on the maternal retrieval response and other aspects of maternal behavior in rats.Behav Neurosci. 2005 Dec;119(6):1588-604. |
| Dopamine D2 receptor agonist (Bromocriptine; suppression of endogenous PRL secretion; early pregnancy) | delayed onset of maternal behaviour; deficit pup retrieval and crouching | Rats | Bridges RS, Ronsheim PM. Prolactin (PRL) regulation of maternal behavior in rats: Bromocriptine treatment delays and PRL promotes the rapid onset of behavior. Endocrinology. 1990;126(2):837–848. |
| Dopamine D2 receptor agonist (Bromocriptine; suppression of endogenous PRL secretion; early pregnancy) | novel cage: pup retrieval deficit, increased anxiety (no change in home cage) | Mice | Larsen CM, Grattan DR. Prolactin-induced mitogenesis in the subventricular zone of the maternal brain during early pregnancy is essential for normal postpartum behavioral responses in the mother. Endocrinology. 2010 Aug;151(8):3805-14. |
| Dopamine D2 receptor agonist (Bromocriptine; suppression of endogenous PRL secretion; early pregnancy) | novel cage: reduced nursing; delayed retrieval; less time on nest; delayed nursing; anxiety normal (no change in home cage) | Rat | Price AK, Bridges RS. The effects of bromocriptine treatment during early pregnancy on postpartum maternal behaviors in rats. Dev Psychobiol. 2014 Sep;56(6):1431-7. |
| Dopamine D2 receptor antagonist (haloperidol; lactation) | deficit pup retrieval; deficit nest building; deficit grooming; **increased nursing** | Rat | Li M. Antipsychotic Drugs on Maternal Behavior in Rats. Behav Pharmacol. 2015 September ; 26(6): 616–626 |
| Dopamine D2 receptor antagonist (clebopride ; nucleus accumbens; pregnant) | delayed retrieval; | Rat | Byrnes, EM, Rigero BA, Bridges, RS. Dopamine antagonists during parturition disrupt maternal care and the retention of maternal behavior in rats. Pharmacol Biochem Behav. 2002 Nov;73(4):869-75. |
| Estradiol  (implant; MPOA; ovariectomised virgin; foster pups) | **stimulates the onset of maternal behaviour** | Rat | Fahrbach SE, Pfaff DW.Effect of preoptic region implants of dilute estradiol on the maternal behavior of ovariectomized, nulliparous rats.Horm Behav. 1986 Sep;20(3):354-63. |
| Estradiol  (implant; progesterone withdrawal; virgin; foster pups) | **stimulates the onset of maternal behaviour** | Rat | Bridges RS. A quantitative analysis of the roles of dosage, sequence, and duration of estradiol and progesterone exposure in the regulation of maternal behavior n the rat.Endocrinology. 1984 Mar;114(3):930-40. |
| Estradiol  (systemic; progesterone withdrawal; ovariectomised virgin; foster pups) | **stimulates the onset of maternal behaviour;** delayed pup retrieval; nest building deficit; increased anxiety | Mice | Murakami G. Distinct Effects of Estrogen on Mouse Maternal Behavior: The Contribution of Estrogen Synthesis in the Brain. PLoS One. 2016 Mar 23;11(3):e0150728 |
| Estrogen synthesis block  (letrozole) | nest building deficit; pup retrieval deficit; nursing ok | Mice | Murakami G. Distinct Effects of Estrogen on Mouse Maternal Behavior: The Contribution of Estrogen Synthesis in the Brain. PLoS One. 2016 Mar 23;11(3):e0150728 |
| Estrogen receptor alpha +ve MPOA optogenetic stimulation  (virgin; foster pups) | **stimulates pup retrieval and pup sniffing** | Mice | Fang YY, Yamaguchi Y, Song SC, Tritsch NX and Lin D. A Hypothalamic Midbrain Pathway Essential for Driving Maternal Behaviors. Neuron 2018 April; 98: 192–207. |
| Gamma-Aminobutyric acid (GABA)(A) receptor agonist  (Benzodiazepine and buspirone; IP; postpartum) | nest building deficit; pup retrieval deficit; decreased aggression | Rat | Ferreira A, Picazo O, Uriarte N, Pereira M, Fernández-Guasti A. Inhibitory effect of buspirone and diazepam, but not of 8-OH-DPAT, on maternal behavior and aggression. Pharmacol Biochem Behav. 2000 Jun;66(2):389-96 |
|  |  |  |  |
| Gamma-Aminobutyric acid (GABA)(A) receptor antagonist  (bicuculline; postpartum) | **increased licking/grooming;** decreased aggression | Mice | Lee G, Gammie SC. GABA(A) receptor signaling in the lateral septum regulates maternal aggression in mice Behav Brain Res. 2010 Dec 1;213(2):230-7 |
| Hypocretin  (ICV; high dose; postpartum) | decreased aggression; delayed latency to nurse; reduced time nursing (arched) |  | D'Anna KL, Gammie SC.Hypocretin-1 dose-dependently modulates maternal behaviour in mice.J Neuroendocrinol. 2006 Aug;18(8):553-66. |
| Hypocretin  (ICV; low dose; postpartum) | **increased licking and grooming; no change in self grooming** | Mice | D'Anna KL, Gammie SC.Hypocretin-1 dose-dependently modulates maternal behaviour in mice.J Neuroendocrinol. 2006 Aug;18(8):553-66. |
| IGF-I infusion/antagonism of brain IGFBP-3 | pup retrieval deficit | Rat | Lékó AH, Cservenák M, Szabó ÉR, Hanics J, Alpár A, Dobolyi Á. Insulin-like growth factor I and its binding protein-3 are regulators of lactation and maternal responsiveness. Sci Rep. 2017 Jun 13;7(1):3396. |
| Methylazoxymethanol  (mitotic inhibitor; early pregnancy) | pup retrieval deficit; increased anxiety | Mice | Larsen & Grattan (2010). Prolactin-induced mitogenesis in the subventricular zone of the maternal brain during early pregnancy is essential for normal postpartum behavioral responses in the mother.Endocrinology 151(8): 3805-3814 |
| Oxytocin  (ICV; virgin ovariectomized and primed with estrogen; foster pups) | **stimulates onset of maternal behaviour** | Rat | Pedersen, CA and Prange AJ. Induction of maternal behavior in virgin rats after intracerebroventricular administration of oxytocin. Proc Natl Acad Sci U S A. 1979 Dec;76(12):6661-5 |
| Oxytocin  (ICV; virgin; foster pups) | **stimulates onset of maternal behaviour** | Rat | Fahrbach SE, Morrell JI, Pfaff DW. Horm Behav. 1984 Sep;18(3):267-86.Oxytocin induction of short-latency maternal behavior in nulliparous, estrogen-primed female rats. |
| Oxytocin  (intranasal; foster pups) | **stimulates onset of maternal behaviour** | Rat | Parreiras-E-Silva LT, Vargas-Pinilla P, Duarte DA, Longo D, Espinoza Pardo GV, Dulor Finkler A, Paixão-Côrtes VR, Paré P, Rovaris DL, Oliveira EB, Caceres RA, Gonçalves GL, Bouvier M, Salzano FM, Lucion AB, Costa-Neto CM, Bortolini MC. Functional New World monkey oxytocin forms elicit an altered signaling profile and promotes parental care in rats. Proc Natl Acad Sci U S A. 2017 Aug 22;114(34):9044-9049 |
| Oxytocin  (systemic; virgin with/without dams and litters) | **stimulates onset of maternal behaviour** | Rat | Marlin BJ, Mitre M, D'amour JA, Chao MV, Froemke RC. Oxytocin enables maternal behaviour by balancing cortical inhibition. Nature. 2015 Apr 23;520(7548):499-504 |
| Oxytocin antagonist  (d(CH2)5-8-ornithine-vasotocin; ICV; post delivery) | delayed onset of maternal behaviour | Rat | van Leengoed E, Kerker E, Swanson HH. Inhibition of post-partum maternal behaviour in the rat by injecting an oxytocin antagonist into the cerebral ventricles. J Endocrinol. 1987 Feb;112(2):275-82. |
| oxytocin antagonist  (infusion; postpartum) | reduced arched-back nursing | Rat | Bosch OJ, Neumann ID.Brain vasopressin is an important regulator of maternal behavior independent of dams' trait anxiety.Proc Natl Acad Sci U S A. 2008 Nov 4;105(44):17139-44. |
| Oxytocin antagonist  (VTA or MPOA) | deficit in pup retrieval; deficit in nurturing | Rat | Pedersen CA, Caldwell JD, Walker C, Ayers G, Mason GA. Oxytocin activates the postpartum onset of rat maternal behavior in the ventral tegmental and medial preoptic areas. Behav Neurosci. 1994 Dec;108(6):1163-71. |
| Oxytocin or tocinoic acid  (ICV; virgin ovariectomized and primed with estrogen; foster pups) | **stimulates onset of maternal behaviour** | Rat | Pedersen CA, Ascher JA, Monroe YL, Prange AJ. Oxytocin induces maternal behavior in virgin female rats. Science. 1982 May 7;216(4546):648-50 |
| MPOAGalanin ablation  (virgin; foster pups) | decreased retrieval behaviour; increased in pup-directed aggression | Mice | Wu Z, Autry AE, Bergan JF, Watabe-Uchida M, Dulac CG. Galanin neurons in the medial preoptic area govern parental behaviour. Nature. 2014; 509:325–330 |
| Optogenetic activation of MPOA^Galanin^ to PAG projections (virgin; foster pups) | **increased pup grooming and sniffing** | Mice | Kohl J, Babayan BM, Rubinstein ND, Autry AE, Marin-Rodriguez B, Kapoor V, Miyamishi K, Zweifel LS, Luo L, Uchida N, Dulac C. Functional circuit architecture underlying parental behaviour. Nature 2018 |
| Optogenetic activation of MPOA^Gal^ to VTA  (virgin; foster pups) | **increased motivation to interact with pups** |  |  |
| Optogenetic activation of MPOA^Gal^ to MeA  (virgin; foster pups) | decreased time in nest |  |  |
| optogenetic stimulation of Esr1+ neurons in MPOA | **induced pup retrieval** |  |  |
| optogenetic ablation of Esr1+ neurons in MPOA | impaired pup retrieval; pup contact and aggression unaffected |  |  |
| Pheromones (male)/virgin  (foster pups) | **stimulates onset of maternal behaviour** | Mice | Larsen CM, Kokay IC, Grattan DR. Male pheromones initiate prolactin-induced neurogenesis and advance maternal behavior in female mice. Horm Behav. 2008 Apr;53(4):509-17 |
| Pheromones (unfamiliar female; pregnant) | increased anxiety; impaired maternal behaviour | Mice | Larsen CM, Grattan DR. Exposure to female pheromones during pregnancy causes postpartum anxiety in mice.Vitam Horm. 2010;83:137-49 |
| Blood transfusion from late pregnant female to virgin | **stimulates onset of maternal behaviour** | Rat | Terkel J. Rosenblatt JS. Humoral factors underlying maternal behavior at parturition: cross transfusion between freely moving rats. J Comp Physiol Psychol. 1972 1972 Sep;80(3):365-71. |
| Placental lactogen  (infusion; ovariectomised; virgin; P followed by E2 + bromocriptine; foster pups) | **stimulates onset of maternal behaviour** | Rat | Bridges RS, Robertson MC, Shiu RPC, Friesen HG, Stuer AM, Mann PE. Endocrine communication between conceptus and mother: placental lactogen stimulation of maternal behavior. Neuroendocrinology. 1996; 64:57–64. |
| Progesterone receptor antagonist (Mifepristone; in pregnancy) | Pup retrieval deficit; maternal rejection | Mice | Wang MW, Crombie DL, Hayes JS, Heap RB. Aberrant maternal behaviour in mice treated with a progesterone receptor antagonist during pregnancy. J Endocrinol. 1995 May;145(2):371-7. |
| Prolactin  (infusion; MPOA; virgin; foster pups) | **stimulates onset of maternal behaviour** | Rat | Bridges RS, Numan M, Ronsheim PM, Mann PE, Lupini CE. Central prolactin infusions stimulate maternal behavior in steroid-treated, nulliparous female rats. Proc Natl Acad Sci USA. 1990;87:8003–8007 |
| Prolactin  (infusion; virgin) | **stimulates onset of maternal behaviour; pup retrieval and crouching** | Rat | Bridges RS, DiBiase R, Loundes DD, Doherty PC. Prolactin stimulation of maternal behavior in female rats. Science. 1985;227:782–78 |
| Prolactin receptor antagonist  (S179D-PRL; lateral ventricle or MPOA; foster pups) | delayed onset of maternal behaviour | Rat | Bridges R, Rigero B, Byrnes E, Yang L, Walker A. Central infusions of the recombinant human prolactin receptor antagonist, S179D-PRL, delay the onset of maternal behavior in steroid-primed, nulliparous female rats. Endocrinology 142, 730 (2001). |
| Prostagandin  (ICV; virgin ovariectomized and primed with estrogen; foster pups) | **stimulates onset of partial maternal behaviour; short term** | Rat | Pedersen CA, Ascher JA, Monroe YL, Prange AJ. Oxytocin induces maternal behavior in virgin female rats. Science. 1985 May 7;216(4546):648-50 |
| Serotinin receptor anatagonist  (quipazine) | decreased aggression | Rat | Olivier B, Mos J, van Oorschot R, Hen R. Serotonin receptors and animal models of aggressive behavior. Pharmacopsychiatry. 1995; 28(Suppl 2):80–90 |
| Serotinin receptor anatagonists (clozapine, risperidone, olanzapine and quetiapine) | deficit pup retrieval; deficit nest building; deficit grooming; decreased nursing (temporal differences) | Rat | Li M. Antipsychotic Drugs on Maternal Behavior in Rats. Behav Pharmacol. 2015 September ; 26(6): 616–626 |
| Serotonin receptor agonist TCB-2 | pup retrieval deficit | Rat | Gao J, Wu R, Davis C, Li M. Activation of 5-HT2A receptor disrupts rat maternal behavior.Neuropharmacology. 2018 Jan;128:96-105 |
| Stress  (in pregnancy; light/heat/noise) | decreased maternal care only with non stressed fostered pups | Mice | Meek, L. R., Dittel, P. L., Sheehan, M. C., Chan, J. Y. & Kjolhaug, S. R. Effects of stress during pregnancy on maternal behavior in mice. Physiol Behav. 72, 473–479 (2001). |
| Stress  (in pregnancy; restraint) | decreased arched-back nursing | Rat | Smith, J. W., Seckl, J. R., Evans, A. T., Costall, B. & Smythe, J. W. Gestational stress induces post-partum depression-like behaviour and alters maternal care in rats. Psychoneuroendocrinology 29, 227–244 (2004). |
| Stress  (in pregnancy; restraint) | increased anxiety; decreased licking; decreased hovering | Rat | Baker, S. et al. Effects of gestational stress: 1. Evaluation of maternal and juvenile offspring behavior. Brain Res. 1213, 98–110 (2008). |
| Stress  (in pregnancy; restraint) | decreased licking and grooming (high LG dams only) | Rat | Champagne, F. A. & Meaney, M. J. Stress during gestation alters postpartum maternal care and the development of the offspring in a rodent model. Biol. Psychiatry 59, 1227–1235 (2006). |
| Stress  (in pregnancy; ultramild stressors) | impaired defence behaviour | Mice | Pardon, M., Gerardin, P., Joubert, C., Perez-Diaz, F. & Cohen-Salmon, C. Influence of prepartum chronic ultramild stress on maternal pup care behavior in mice. Biol. Psychiatry 47, 858–863 (2000). |
| Stress  (late pregnancy; restraint+light) | decreased duration of pup contact; increased latency and decreased duration of maternal behaviour | Mice | Belnoue L, Malvaut S, Ladevèze E, Abrous DN, Koehl M.Plasticity in the olfactory bulb of the maternal mouse is prevented by gestational stress.Sci Rep. 2016 Nov 25;6:37615. |
| vasopressin  (infusion; postpartum) | **increased arched back nursing** | Rat | Bosch OJ, Neumann ID.Brain vasopressin is an important regulator of maternal behavior independent of dams' trait anxiety.Proc Natl Acad Sci U S A. 2008 Nov 4;105(44):17139-44. |
| vassopressin  (ICV; virgin ovariectomized and primed with estrogen) | **stimulates onset of maternal behaviour (foster pups)** | Rat | Pedersen CA, Ascher JA, Monroe YL, Prange AJ. Oxytocin induces maternal behavior in virgin female rats. Science. 1985 May 7;216(4546):648-50 |
| vasopressin (V1) antagonist  (infusion; postpartum) | reduced arched-back nursing | Rat | Bosch OJ, Neumann ID.Brain vasopressin is an important regulator of maternal behavior independent of dams' trait anxiety.Proc Natl Acad Sci U S A. 2008 Nov 4;105(44):17139-44. |
| vasopressin (V1) antagonist  (infusion; MPOA) | deficit in pup retrieval; deficit in nurturing | Rat | Pedersen CA, Caldwell JD, Walker C, Ayers G, Mason GA. Oxytocin activates the postpartum onset of rat maternal behavior in the ventral tegmental and medial preoptic areas.Behav Neurosci. 1994 Dec;108(6):1163-71. |
| Vasopressin 1b receptor antagonist (mpBNST) | decreased licking/grooming; reduced arched back nursing | Rat | Bayerl DS, Kaczmarek V, Jurek B, van den Burg EH, Neumann ID, Gaßner BM, Klampfl SM, Bosch OJ. Antagonism of V1b receptors promotes maternal motivation to retrieve pups in the MPOA and impairs pup-directed behavior during maternal defense in the mpBNST of lactating rats.Horm Behav. 2016 Mar;79:18-27. |
| Vasopressin 1b receptor antagonist (MPOA) | increased pup retrieval; reduced arched back nursing | Rat | Bayerl DS, Kaczmarek V, Jurek B, van den Burg EH, Neumann ID, Gaßner BM, Klampfl SM, Bosch OJ. Antagonism of V1b receptors promotes maternal motivation to retrieve pups in the MPOA and impairs pup-directed behavior during maternal defense in the mpBNST of lactating rats.Horm Behav. 2016 Mar;79:18-27. |
| Vasopressin 1b receptor antagonist (systemic) | decreased nursing and mother-pup interaction | Rat | Bayerl DS, Klampfl SM, Bosch OJ. Cen+A70:D113tral V1b receptor antagonism in lactating rats: impairment of maternal care but not of maternal aggression.J Neuroendocrinol. 2014 Dec;26(12):918-26. |
