# Supplementary material for: Maternal care boosted by paternal imprinting in mammals
Source: PLoS Biol. 2018 Jul 31;16(7):e2006599. doi: 10.1371/journal.pbio.2006599 (PMC6067684; doi:10.1371/journal.pbio.2006599)
Supplement: S2 Table — Phlda2, pleckstrin homology-like domain family A member 2. (DOCX) [file pbio.2006599.s002.docx]

| **Dam/dosage** | | **Stats** | **WT(0x)**  **± SEM** | **WT(1x)**  **± SEM** | **WT(2x)**  **± SEM** | ***Post Hoc* tests** | | |
| --- | --- | --- | --- | --- | --- | --- | --- | --- |
| Offspring genotype | | ANOVA (unless stated) | *Phlda2*^-/+^ | *Phlda2*^+/+^ | *Phlda2*^+/+BACx1^ | WT(0x) v WT(1x) | WT(1x) v WT(2x) | WT(0x) v WT(2x) |
| Litter size (gene expression) | |  | 7.08 ± 0.35 | 7.14 ± 0.63 | 6.91 ± 0.25 | >0.05 | >0.05 | >0.05 |
| E16.5 fetal weight | | (F2, 167 = 41.7, p **< 0.001**) | 489 ± 7.43 | 579 ± 7.37 | 484 ± 11.57 | **<0.001** | **<0.001** | >0.05 |
| Litter size (behaviour) | | (F2, 36 = 1.0, p = 0.438) | 6.91 ± 0.25 | 7.14 ± 0.64 | 7.08 ± 0.35 | >0.05 | >0.05 | >0.05 |
| P7 pup weight | | (F2, 164 = 1.89, p = 0.15) | 3.75 ± 0.042 | 3.88 ± 0.052 | 3.79 ± 0.065 | >0.05 | >0.05 | >0.05 |
| E0.5 maternal weight | | (F2, 36 = 0.451, p = 0.641) | 22.8 ± 0.4 | 21.9 ± 0.4 | 23.3 ± 0.7 | >0.05 | >0.05 | >0.05 |
| P7 maternal weight | | (F2, 36 = 2.37, p = 0.11) | 29.6 ± 0.8 | 29.5 ± 0.5 | 27.6 ± 0.8 | >0.05 | >0.05 | >0.05 |
| EPM (P2) | duration in open | (F2, 36 = 0.16, p = 0.85) | 24.2 ± 22.5 | 11.2 ± 9.6 | 22.2 ± 17.5 | >0.05 | >0.05 | >0.05 |
|  | duration in middle | (F2, 36 = 1.5, p = 0.24) | 85.3 ± 24.7 | 161.3 ± 30.3 | 109.4 ± 25.9 | >0.05 | >0.05 | >0.05 |
|  | duration in closed | (F2, 36 = 0.80, p = 0.46) | 190.5 ± 29.5 | 127.5 ± 31.3 | 168.5 ± 24.7 | >0.05 | >0.05 | >0.05 |
|  | velocity in open | (F2, 36 = 2.53, p = 0.093) | 7.2 ± 1.8 | 2.9 ± 0.50 | 2.9 ± 0.29 | 0.077 | >0.05 | >0.05 |
|  | velocity in middle | (F2, 36 = 0.75, p = 0.48) | 2.0 ± 0.27 | 1.6 ± 0.26 | 2.0 ± 0.25 | >0.05 | >0.05 | >0.05 |
|  | velocity in closed | (F2, 36 = 2.17, p = 0.13) | 1.75 ± 0.2 | 2.0 ± 0.22 | 2.3 ± 0.31 | >0.05 | >0.05 | >0.05 |
|  | distance travelled in open | (F2, 36 = 54, p = 0.59) | 38.7 ± 23.3 | 25.5 ± 10.0 | 63.8 ± 29.6 | >0.05 | >0.05 | >0.05 |
|  | distance travelled in middle | (F2, 36 = 0.15, p = 0.86) | 140.6 ± 36.1 | 147.1 ± 18.8 | 162.4 ± 26.3 | >0.05 | >0.05 | >0.05 |
|  | distance travelled in closed | (F2, 36 = 0.59, p = 0.56) | 278.5 ± 60.8 | 268.4 ± 49.1 | 304.2 ± 71.3 | >0.05 | >0.05 | >0.05 |
|  | frequency of entry into open | (F2, 36 = 0.94, p = 0.40) | 1.83 ± 0.46 | 1.81 ± 0.64 | 3.1 ± 0.89 | >0.05 | >0.05 | >0.05 |
|  | frequency of entry into middle | (F2, 36 = 1.66, p = 0.21) | 10.7 ± 2.96 | 6.5 ± 1.26 | 11.5 ± 1.60 | >0.05 | >0.05 | >0.05 |
|  | frequency of entry into closed | (F2, 36 = 1.44, p = 0.25) | 8.6 ± 2.97 | 4.3 ± 1.60 | 8.1 ±1.41 | >0.05 | >0.05 | >0.05 |
|  | rearing frequency in open | (F2, 36 = 1.66, p = 0.21) | 1.3 ± 0.56 | 1.2 ± 0.5 | 2.5 ± 0.9 | >0.05 | >0.05 | >0.05 |
|  | rearing frequency in middle | (F2, 36 = 1.66, p = 0.21) | 13.8 ± 4.1 | 6.7 ± 1.41 | 12.5 ± 2.02 | >0.05 | >0.05 | >0.05 |
|  | rearing frequency in closed | (F2, 36 = 1.66, p = 0.21) | 21.5 ± 9.1 | 7.3 ± 2.57 | 24.5 ± 7.56 | >0.05 | >0.05 | >0.05 |
|  | stretch frequency in open | (F2, 36 = 0.65, p = 0.526) | 4.6 ± 3.36 | 1.8 ± 0.67 | 3.6 ± 1.79 | >0.05 | >0.05 | >0.05 |
|  | stretch frequency in middle | (F2, 36 = 0.76, p = 0.474) | 12.3 ± 2.24 | 17.4 ± 2.97 | 17.9 ± 3.31 | >0.05 | >0.05 | >0.05 |
|  | stretch frequency in closed | (F2, 36 = 1.10, p = 0.34) | 7.4 ± 1.53 | 5.8 ± 1.59 | 9.5 ± 1.58 | >0.05 | >0.05 | >0.05 |
|  | dip frequency in open | (F2, 36 = 0.89, p = 0.42) | 1.5 ± 1.18 | 0.8 ± 0.44 | 1.9 ± 0.74 | >0.05 | >0.05 | >0.05 |
|  | dip frequency in middle | (F2, 36 = 2.1, p = 0.14) | 5.5 ± 1.21 | 8.75 ± 1.41 | 10.4 ± 1.89 | >0.05 | >0.05 | >0.05 |
|  | dip frequency in closed | (F2, 36 = 1.1, p = 0.35) | 0.75 ± 0.41 | 0.25 ± 0.11 | 0.9 ± 0.31 | >0.05 | >0.05 | >0.05 |
| Pup retrieval (P3) | latency to sniff | (F2, 36 = 1.1, p = 0.33) | 66.8 ± 8.89 | 49.9 ± 7.49 | 54.7 ± 8.91 | >0.05 | >0.05 | >0.05 |
|  | latency to retrieve | (F2, 36 = 4.76, p = **0.015**) | 339.1 ± 12.7 | 175.8 ± 17.4 | 179.3± 10.7 | **0.01** | >0.05 | 0.066 |
|  | nest  duration | (F2, 36 = 1.57, p = 0.22) | 1742 ± 226 | 1558 ± 188 | 2102 ± 303 | >0.05 | 0.07 | >0.05 |
|  | nest  visits | (F2, 36 = 0.41, p = 0.67) | 3.8 ± 0.56 | 3.19 ± 0.52 | 3.85 ± 0.79 | >0.05 | >0.05 | >0.05 |
|  | food zone  duration | (F2, 36 = 0.69, p = 0.51) | 79.8 ± 67.7 | 130.5 ± 30.1 | 162.2 ± 28.7 | >0.05 | >0.05 | >0.05 |
|  | food zone  visits | (F2, 36 = 0.83, p = 0.44) | 9.9 ± 3.7 | 15.1 ± 2.20 | 14.3 ± 2.5 | >0.05 | >0.05 | >0.05 |
|  | water zone  duration | (F2, 36 = 1.51, p = 0.24) | 7.9 ± 2.71 | 19.2 ± 5.95 | 12.4 ± 2.1 | >0.05 | >0.05 | >0.05 |
|  | water zone  visits | (F2, 36 = 0.263, p = 0.77) | 9.8 ± 2.98 | 12.9 ± 3.0 | 11.6 ± 2.6 | >0.05 | >0.05 | >0.05 |
|  | distance moved | (F2, 36 = 0.91, p = 0.91) | 3121 ± 666 | 2876 ± 278 | 2835 ± 430 | >0.05 | >0.05 | >0.05 |
|  | velocity | (F2, 36 = 0.44, p = 0.65) | 1.59 ± 0.24 | 1.83 ± 0.18 | 1.61 ± 0.21 | >0.05 | >0.05 | >0.05 |
| Nest building (P4) | number of nests built with pups | (F2, 36 = 4.3, p = **0.022**) | 1 | 3 | 7 | >0.05 | **0.05** | **0.021** |
|  | nest building  duration | (F2, 36 = 7.0, p = **0.003**) | 1298 ± 427 | 2414 ± 289 | 2893 ± 108 | **0.029** | >0.05 | **0.002** |
|  | nest zone  duration | (F2, 36 = 7.95, p = 0.505) | 1792 ± 330 | 1385 ± 253 | 1367 ± 279 | >0.05 | >0.05 | >0.05 |
|  | nest zone  visits | (F2, 36 = 0.82, p = 0.49) | 10.6 ± 2.4 | 15.6 ± 1.9 | 16.3 ± 3.3 | >0.05 | >0.05 | >0.05 |
|  | total nursing duration | (F2, 36 = 5.8, p = **0.007**) | 1439 ± 391 | 570 ± 295 | 41 ± 26 | 0.085 | >0.05 | **0.005** |
|  | total nursing events | (F2, 36 = 6.9, p = **0.003**) | 8.7 ± 1.93 | 3.2 ± 1.19 | 1.7 ± 0.84 | **0.016** | >0.05 | **0.003** |
|  | crouched nursing duration | (F2, 36 = 6.5, p = **0.004**) | 1018 ± 334 | 356 ± 215 | 9.61 ± 7.72 | **0.05** | >0.05 | **0.009** |
|  | crouched nursing events | (F2, 36 = 5.0, p = **0.012**) | 3.6 ± 1.01 | 1.19 ± 0.24 | 0.38 ± 0.24 | **0.02** | >0.05 | **0.003** |
|  | arched nursing duration | (F2, 36 = 3.1, p = 0.059) | 169.3 ± 80.5 | 64 ± 31.68 | 21.10 ± 13.30 | >0.05 | >0.05 | 0.07 |
|  | arched nursing events | (F2, 36 = 2.7, p = 0.08) | 3.4 ± 0.81 | 1.63 ± 0.56 | 1.15 ± 0.55 | >0.05 | >0.05 | 0.058 |
|  | passive nursing duration | (F2, 36 = 1.1, p = 0.347) | 252.2 ± 115.60 | 150 ± 135.86 | 10.46 ± 7.11 | >0.05 | >0.05 | >0.05 |
|  | passive nursing events | (F2, 36 = 4.7, p = **0.016**) | 1.7 ± 0.72 | 0.38 ± 0.22 | 0.15 ± 0.10 | **0.039** | >0.05 | **0.019** |
|  | contact with pups duration | (F2, 36 = 2.83, p = 0.072) | 29.9 ± 7.41 | 49.88 ± 8.36 | 26.54 ± 6.89 | >0.05 | >0.05 | >0.05 |
|  | contact with pups events | (F2, 36 = 2.23, p = 0.12) | 3.8 ± 0.8 | 6.3 ± 0.91 | 4.45 ± 0.85 | >0.05 | >0.05 | >0.05 |
|  | pup grooming  duration | (F2, 36 = 4.4, p = **0.020**) | 58.7 ± 26.59 | 17.0 ± 3.27 | 7.07 ± 3.33 | 0.057 | >0.05 | **0.02** |
|  | pup grooming events | (F2, 36 = 4.6, p = **0.017**) | 2.8 ± 0.81 | 1.69 ± 0.28 | 0.76 ± 0.28 | >0.05 | >0.05 | **0.012** |
|  | self-grooming duration | (F2, 36 = 6.6, p = **0.004**) | 53.5 ± 14.72 | 17.56 ± 7.47 | 6.77 ± 4.41 | **0.021** | >0.05 | **0.003** |
|  | self grooming events | (F2, 36 = 5.0, p = **0.012**) | 1.9 ± 0.55 | 0.88 ± 0.34 | 0.23 ± 0.12 | **0.02** | >0.05 | **0.01** |
|  | food zone  duration | (F2, 36 = 3.58, p = 0.78) | 159 ± 38 | 110 ± 36 | 126 ± 43 | >0.05 | >0.05 | >0.05 |
|  | food hopper  visits | (F2, 36 = 5.49, p = **0.003**) | 42.6 ± 10.26 | 10.1 ± 2.50 | 15.0 ± 4.01 | **0.001** | >0.05 | **0.02** |
|  | water zone  duration | (F2, 36 = 0.66, p = 0.58) | 9.9 ± 6.83 | 3.3 ± 1.51 | 3.5 ± 2.02 | >0.05 | >0.05 | >0.05 |
|  | water zone  visits | (F2, 36 = 0.92, p = 0.44) | 7.8 ± 5.05 | 2.3 ± 0.8 | 2.1 ± 0.98 | >0.05 | >0.05 | >0.05 |
|  | distance travelled | (F2, 36 = 5.78, p = **0.002**) | 5614 ± 348 | 3184 ± 316 | 3607 ± 596 | **0.001** | >0.05 | **0.001** |
|  | velocity | (F2, 36 = 0.89, p = 0.46) | 2.7 ± 0.37 | 2.8 ± 0.25 | 3.4 ± 0.41 | >0.05 | >0.05 | >0.05 |
| 23 hrs - overall | nest duration | (F2, 36 = 4.63, p = **0.016**) | 51576 ± 2585 | 47930 ± 2255 | 54364 ± 2218 | >0.05 | **0.014** | >0.05 |
|  | nest frequency | (F2, 36 = 3.7, p = **0.035**) | 95.1 ± 5.18 | 84.4 ± 3.61 | 98.3 ± 5.62 | >0.05 | **0.031** | >0.05 |
|  | food duration | (F2, 36 = 3.10, p = 0.057) | 9739 ± 987 | 9798 ± 735 | 8265 ± 823 | >0.05 | >0.05 | >0.05 |
|  | food frequency | (F2, 36 = 1.149, p = 0.328) | 363.5 ± 43.2 | 361.8 ± 26.5 | 628.2 ± 30.8 | >0.05 | >0.05 | >0.05 |
|  | water duration | (F2, 36 = 0.80, p = 0.46) | 369.2 ± 105.8 | 865.0 ± 597 | 328.7 ± 88.72 | >0.05 | >0.05 | >0.05 |
|  | water frequency | (F2, 36 = 1.469, p = 0.244) | 279.0 ± 64.5 | 223.8 ± 36.3 | 208.5 ± 71 | >0.05 | >0.05 | >0.05 |
|  | distance moved | (F2, 36 = 5.1, p = 0.608) | 39542 ± 3562 | 39192 ± 2701 | 36022 ± 3512 | >0.05 | >0.05 | >0.05 |
|  | velocity | (F2, 36 = 0.32, p = 0.73) | 0.8 ± 0.06 | 0.9 ± 0.06 | 0.9 ± 0.03 | >0.05 | >0.05 | >0.05 |
| Phase affects – light/dark | effect of phase on activity/distance | General linear Model | Light =1468.9 ± 164.3  Dark =1655.4 ± 169.0 | Light = 1470.9 ± 131.2  Dark =1917.7 ± 149.0 | Light = 1803.2 ± 220.1  Dark =1717.6 ± 105.9 | p = **0.038**  WT(0x)  p = 0.832 | p **< 0.001** | >0.05  WT(2x)  p = 0.126 |
|  | distance travelled in light phase | (F2, 36 = 1.2, p = 0.31) | 1468.9 ± 164.3 | 1470.9 ± 131.2 | 1803.2 ± 220.1 | >0.05 | >0.05 | >0.05 |
|  | distance travelled in dark phase | (F2, 36 = 0.92, p = 0.41) | 1655.4 ± 169.0 | 1917.7 ± 149.0 | 1717.6 ± 105.9 | >0.05 | >0.05 | >0.05 |
|  | time spent in nest in light phase | (F2, 36 = 0.29, p = 0.75) | 1019.6 ± 126.5 | 912.5 ± 128.7 | 881.7 ± 103.4 | >0.05 | >0.05 | >0.05 |
|  | time spent in nest in dark phase | (F2, 36 = 6.06, p = **0.006**) | 1120.2 ± 199.1 | 455.8 ± 98.4 | 884.0 ± 145.1 | **0.005** | >0.05 | >0.05 |
|  | time spent in food zone in light phase | (F2, 36 = 1.53, p = 0.23) | 288.7 ± 44.6 | 310.4 ± 29.9 | 408.8 ± 77.2 | >0.05 | >0.05 | >0.05 |
|  | time spent in food zone in light phase | (F2, 36 = 2.01, p = 0.15) | 424.1 ± 52.20 | 532.0 ± 40.0 | 445.4 ± 27.7 | >0.05 | >0.05 | >0.05 |
| Am-pm (4 hr transition) | time spent in nest | (F2, 36 = 4.63, p = **0.016**) | 10391.9 ± 528.0 | 8603.8 ± 768.1 | 11306.5 ± 559.9 | >0.05 | **0.014** | >0.05 |
|  | number of visits to nest | (F2, 36 = 3.70, p = **0.035**) | 18.7 ± 1.27 | 15.44 ± 1.55 | 20.23 ± 1.00 | >0.05 | **0.031** | >0.05 |
|  | time spent in food zone | (F2, 36 = 3.10, p = 0.057) | 2519.7 ± 192.0 | 3049.5 ± 267.0 | 2241.3 ± 284.9 | >0.05 | 0.051 | >0.05 |
|  | number of visits to food zone | (F2, 36 = 3.35, p = **0.046**) | 75.8 ± 10.85 | 108.69 ± 15.76 | 151.5 ± 10.8 | >0.05 | 0.056 | >0.05 |
|  | distance moved | (F2, 36 = 0.51, p = 0.61) | 9525 ± 790.1 | 10427.7 ± 991.6 | 9080 ± 967.7 | >0.05 | >0.05 | >0.05 |
|  | water duration | (F2, 36 = 0.80, p = 0.46) | 110.6 ± 15.9 | 100.1 ± 20.0 | 63.8 ± 28.5 | >0.05 | >0.05 | >0.05 |
|  | water frequency | (F2, 36 = 1.47, p = 0.24) | 71.3 ± 9.8 | 68.8 ± 14.0 | 71.3 ± 23.5 | >0.05 | >0.05 | >0.05 |
|  | velocity | (F2, 36 = 0.32, p = 0.73) | 0.91 ± 0.06 | 1.0 ± 0.07 | 0.96 ± 0.06 | >0.05 | >0.05 | >0.05 |
| Food consumed | | (F2, 36 = 3.33, p = **0.05**) | 15.16 ± 1.01 | 17.32 ± 1.2 | 14.65 ± 0.99 | >0.05 | **0.036** | >0.05 |
| Water consumed | | (F2, 36 = 3.04, p = 0.06) | 14.8 ± 1.03 | 17.3 ± 0.99 | 16.1 ± 0.96 | >0.05 | >0.05 | >0.05 |
| USV | P2 | T-TEST | 480.7 ± 76.6 | 508.6 ± 48.6 |  | >0.05 | >0.05 | >0.05 |
|  | P4 | T-TEST | 380.8 ± 155.5 | 637.7 ± 104.7 |  | >0.05 | >0.05 | >0.05 |
