# Supplementary material for: Maternal care boosted by paternal imprinting in mammals
Source: PLoS Biol. 2018 Jul 31;16(7):e2006599. doi: 10.1371/journal.pbio.2006599 (PMC6067684; doi:10.1371/journal.pbio.2006599)
Supplement: S3 Table — Phlda2, pleckstrin homology-like domain family A member 2; WT, wild-type. (DOCX) [file pbio.2006599.s003.docx]

| **Dams genotype/dosage** | | **ANOVA** | **WT (1x)wt**  **± SEM**  **n = 15** | **WT(0x)wt**  **± SEM**  **n = 15** |
| --- | --- | --- | --- | --- |
| Offspring prenatal genotype | |  | WT (*Phlda2*^+/+^) | KO (*Phlda2*^-/+^) |
| Offspring genotype post fostering | |  | WT (*Phlda2*^+/+^) | WT (*Phlda2*^+/+^) |
| Litter size (born)  (6 WT pups fostered to dam) | | (F1, 30 = 0.49, p = 0.49) | 8.6 ± 0.42 | 7.7 ± 0.53 |
| WT pup weight P0 | | (F1, 364 = 0.33, p = 0.57) | 1.35 ± 0.01 | 1.36 ± 0.01 |
| WT pup weight P21 | | (F1, 191 = 0.024, p = 0.88) | 10.89 ± 0.13 | 10.86 ± 0.07 |
| Pup retrieval (P3) | latency to sniff | (F1, 30 = 0.08, p = 0.78) | 19.7 ± 7.7 | 17.0 ± 5.7 |
|  | latency to retrieve | (F1, 30 = 4.04, p = 0.054) | 342.7 ± 78.4 | 160.3 ± 45.7 |
|  | nest  duration | (F1, 30 = 0.23, p = 0.64) | 532.6 ± 181.0 | 684.5 ± 263.3 |
|  | nest zone  visits | (F1, 30 = 0.15, p = 0.71) | 79.5 ±13.6 | 86.4 ± 12.1 |
|  | food zone  duration | (F1, 30 = 0.40, p = 0.53) | 120.5 ± 79.1 | 65.65 ± 35.6 |
|  | food zone  visits | (F1, 30 = 0.053, p = 0.82) | 10.6 ± 2.4 | 9.9 ± 2.4 |
|  | water zone  duration | (F1, 30 = 0.88, p = 0.36) | 63.0 ± 42.6 | 22.8 ± 4.1 |
|  | water zone  visits | (F1, 30 = 0.69, p = 0.41) | 10.2 ± 2.2 | 12.5 ± 1.8 |
|  | distance moved | (F1, 30 = 0.42, p = 0.53) | 2042.9 ± 234.6 | 2357.3 ± 427.6 |
|  | velocity | (F1, 30 = 0.55, p = 0.46) | 2.6 ± 0.25 | 2.9 ± 0.3 |
| Nest building (P4) | number of nests built with pups | Chi-Squared  Chi = 4.65, df = 1, p = **0.03** | 7 (43.8%) | 13 (81.3%) |
|  | nest building  duration | (F1, 30 = 0.008, *p* = 0.93) | 1975.8 ± 279.5 | 1806 ± 233.6 |
|  | Nest building  events | (F1, 30 = 0.12, *p* = 0.73) | 2.63 ± 0.39 | 3.25 ± 0.49 |
|  | nest zone  duration | (F1, 30 = 0.008, p = 0.93) | 1258.7 ± 187.4 | 1277.3 ± 196.8 |
|  | nest zone  visits | (F1, 30 = 0.12, p = 0.73) | 194.1 ± 28.2 | 213.94 ± 34.9 |
|  | total nursing duration | (F1, 30 = 0.87, p = 0.360) | 1654.4 ± 288.0 | 1262.2 ± 308.2 |
|  | total nursing events | (F1, 30 = 4.33, p = **0.046**) | 5.9 ± 0.9 | 3.6 ± 0.7 |
|  | crouched nursing duration | (F1, 30 = 2.0, p = 0.17) | 953.4 ± 181.1 | 616.3 ± 154.1 |
|  | crouched nursing events | (F1, 30 = 0.15, p = 0.71) | 1.9 ± 0.3 | 1.8 ± 0.4 |
|  | arched nursing duration | (F1, 30 = 3.23, p = 0.082) | 212.9 ± 67.4 | 75.5 ± 36.2 |
|  | arched nursing events | (F1, 30 = 8.58, p = **0.006**) | 1.9 ± 0.4 | 0.56 ± 0.2 |
|  | passive nursing duration | (F1, 30 = 0.19, p = 0.67) | 488.1 ± 119.0 | 570.3 ± 149.1 |
|  | passive nursing events | (F1, 30 = 2.83, p = 0.10) | 2.1 ± 0.4 | 1.3 ± 0.3 |
|  | contact with pups events | (F1, 30 = 16.9, p < **0.001**) | 4.1 ± 0.5 | 1.8 ± 0.3 |
|  | pup grooming  duration | (F1, 30 = 6.74, p = **0.014**) | 116.5 ± 18.9 | 58.7 ± 11.8 |
|  | pup grooming events | (F1, 30 = 5.0, p = **0.033**) | 3.0 ± 0.4 | 1.6 ± 0.3 |
|  | self-grooming duration | (F1, 30 = 0.72, p = 0.404) | 61.5 ± 17.4 | 41.3 ± 16.3 |
|  | self grooming events | (F1, 30 = 0.11, p = 0.74) | 1.8 ± 0.3 | 1.6 ± 0.5 |
|  | food zone  duration | (F1, 30 = 1.9, p = 0.18) | 75.6 ± 29.6 | 130.4 ± 29.6 |
|  | food hopper  visits | (F1, 30 = 0.24, p = 0.63) | 10.6 ± 2.0 | 11.9 ± 2.3 |
|  | water zone  duration | (F1, 30 = 0.13, p = 0.72) | 14.9 ± 7.1 | 10.5 ± 5.5 |
|  | water zone  visits | (F1, 30 = 0.053, p = 0.82) | 3.3 ± 0.9 | 3.9 ± 0.9 |
|  | distance travelled | (F1, 30 = 0.005, p = 0.94) | 3825.8 ± 531.0 | 4022.2 ± 360.7 |
|  | velocity | (F1, 30 = 0.15, p = 0.70) | 2.00 ± 0.2 | 2.1 ± 0.2 |
| 23 hrs - overall | nest frequency | (F1, 30 = 0.03, p = 0.87) | 758.6 ± 94.1 | 778.6 ± 75.2 |
|  | nest duration | (F1, 30 = 0.72, p = 0.40) | 10092.1 ± 2391.4 | 12807.6 ± 2152.8 |
|  | food duration | (F1, 30 = 0.58, p = 0.45) | 5806.8 ± 984.1 | 4909.9 ± 686.3 |
|  | food frequency | (F1, 30 = 5.1, p = **0.03**) | 411.1 ± 39.9 | 307.7 ± 24.6 |
|  | water duration | (F1, 30 = 0.38, p = 0.54) | 1731.2 ± 519.1 | 1415.7 ± 126.3 |
|  | water frequency | (F1, 30 = 0.68, p = 0.42) | 189.7 ± 28.9 | 163.5 ± 15.1 |
|  | distance moved | (F1, 30 = 1.29, p = 0.26) | 24691 ± 1656 | 29961 ± 2499 |
|  | velocity | (F1, 30 = 0.00, p = 0.99) | 1.2 ± 0.1 | 1.2 ± 0.1 |
