# Supplementary figures and images for: Maternal care boosted by paternal imprinting in mammals
Source: PLoS Biol. 2018 Jul 31;16(7):e2006599. doi: 10.1371/journal.pbio.2006599 (PMC6067684; doi:10.1371/journal.pbio.2006599)

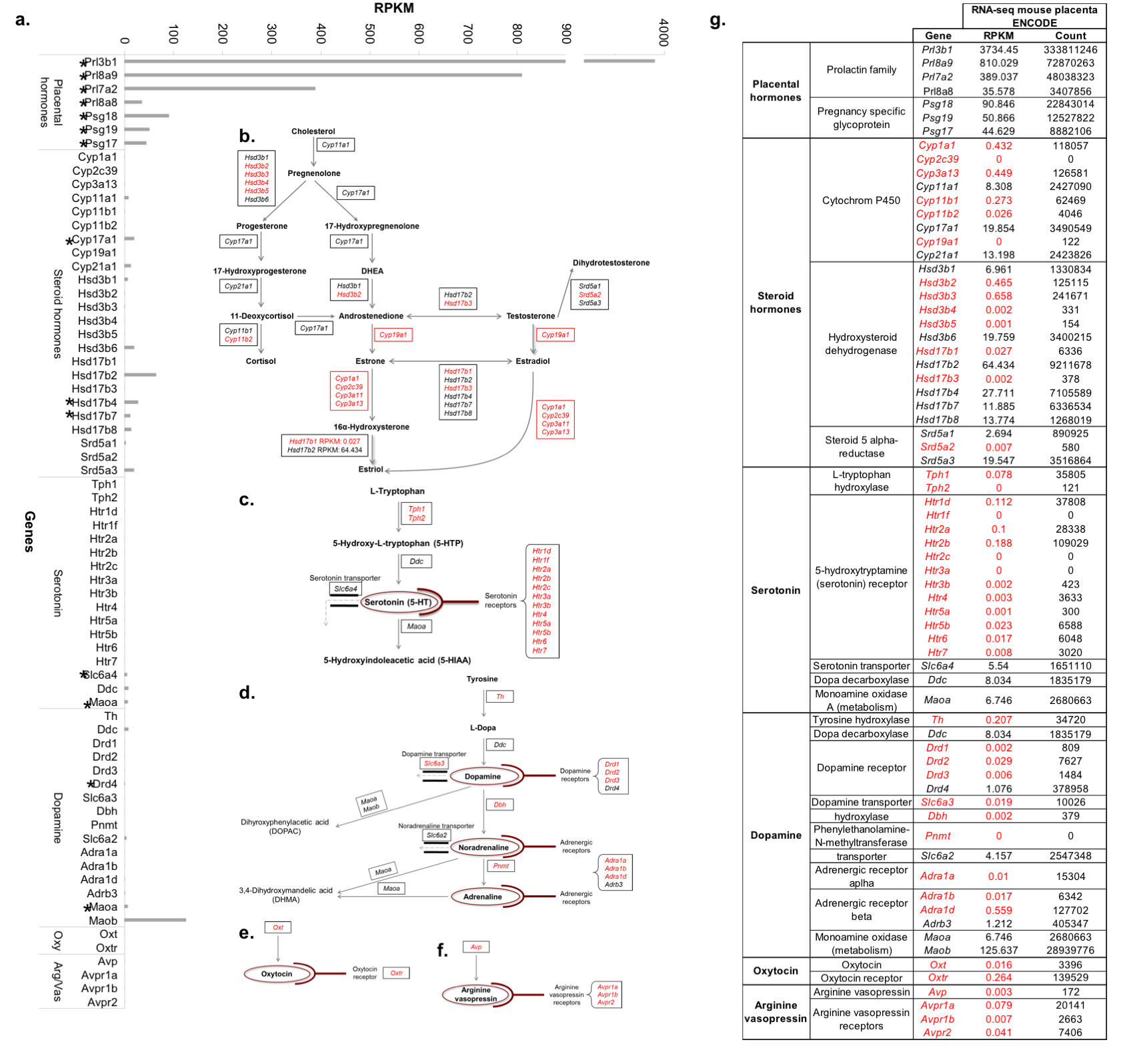

Supplement: S1 Fig — Mouse ENCODE transcriptome data. In red, genes with a lower RPKM value than 1 (genes that are not expressed in placenta); in black, genes with a higher RPKM value than 1. (A) Graph representation of gene expression levels of the different pathways represented plus gene expression levels of placental genes. (B) Steroid hormones. (C) Serotonin. (D) Dopamine, noradrenaline, adrenaline. (E) Oxytocin (labelled ‘Oxy’). (F) Arginine vasopressin (labelled ‘Arg/Vas’). *Altered in Phlda2 mutant placenta. (G) Expression levels in mature mouse placenta. Mouse ENCODE transcriptome data. Count: approximate total reads mapped to gene transcript features. In red genes with a lower RPKM value than 1 (genes that are not expressed in placenta), in black genes with a higher RPKM value than 1. Phlda2, pleckstrin homology-like domain family A member 2; RPKM, reads per kilobase per million reads placed. (TIFF) [file pbio.2006599.s004.tiff]

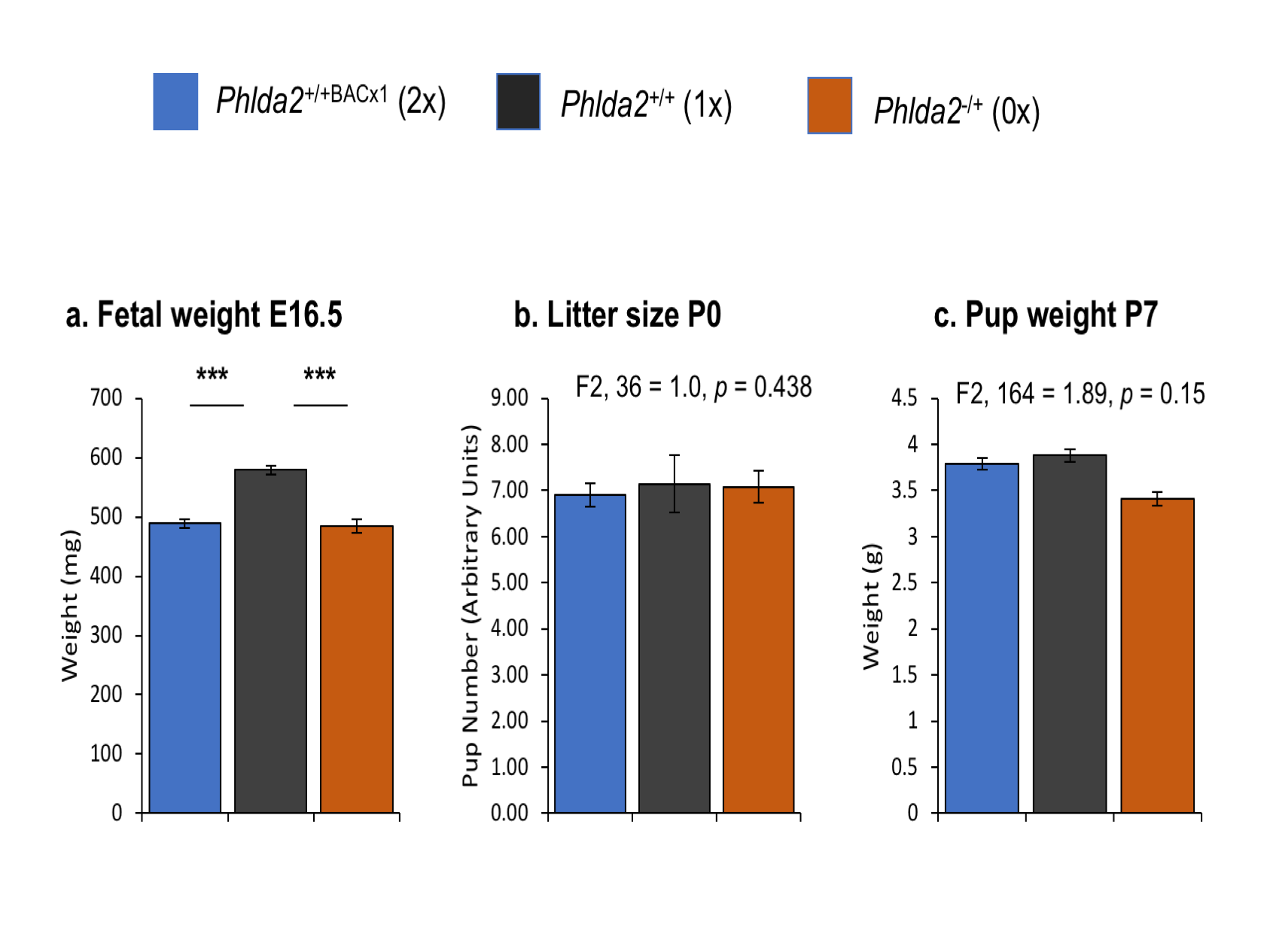

Supplement: S2 Fig — (A) Significant difference in foetal weight at E16.5 across the three models (F2, 254 = 4.7, p = 0.045). (B) No difference in litter size across three models (F2, 36 = 0.83, p = 0.45). (C) Adequate catch-up growth of both Phlda2−/+ and Phlda2+/+BACx1 pups by P7. Numerical data can be found at https://osf.io/543jg/ “RAW NUMERICAL DATA.xlsx”, Sheets labelled Litter_Size_Weight. E, embryonic day; P, postnatal day. (TIFF) [file pbio.2006599.s005.tiff]

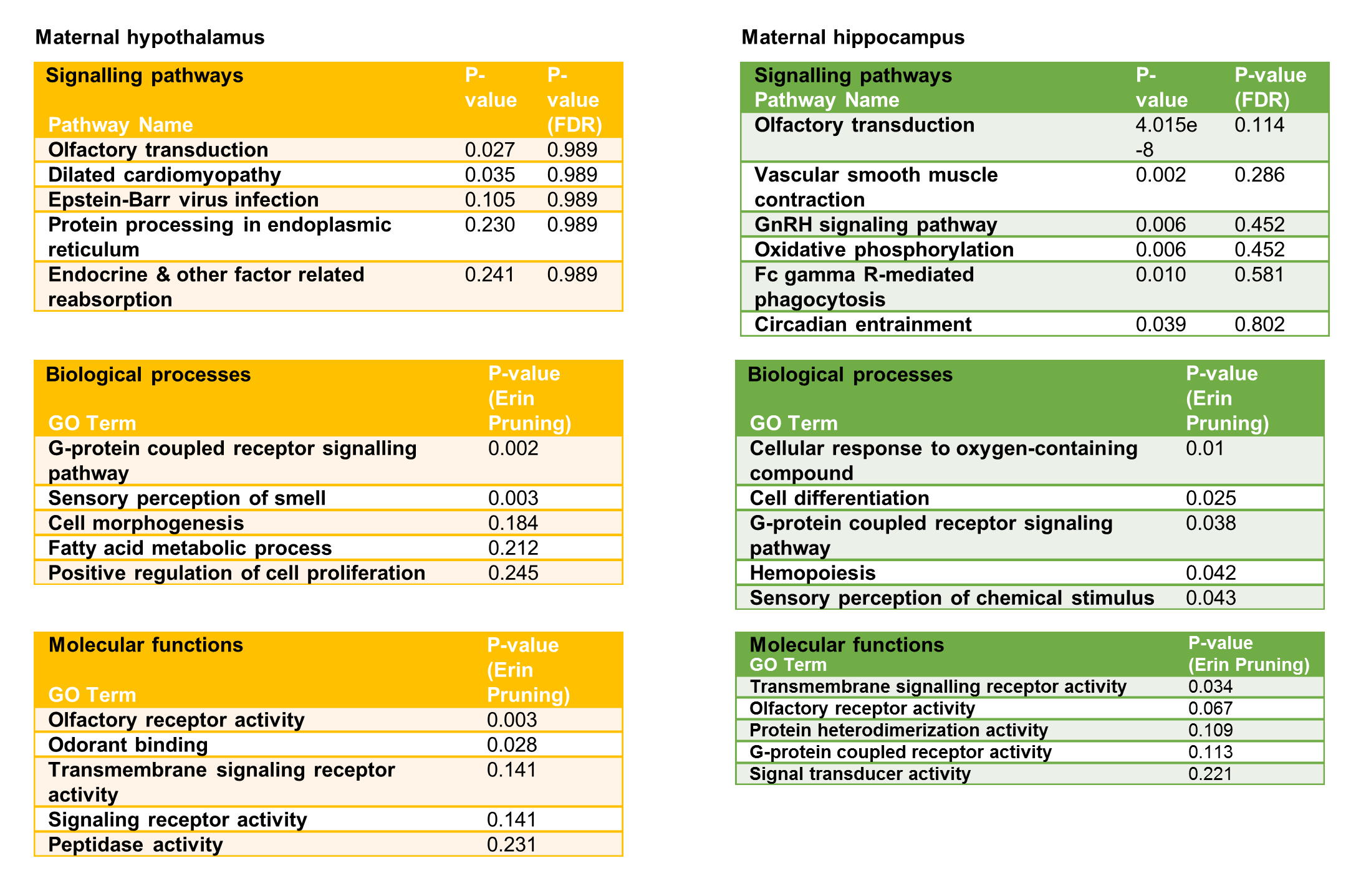

Supplement: S3 Fig — Raw data can be found using accession number: GSE115276. (TIF) [file pbio.2006599.s006.tif]

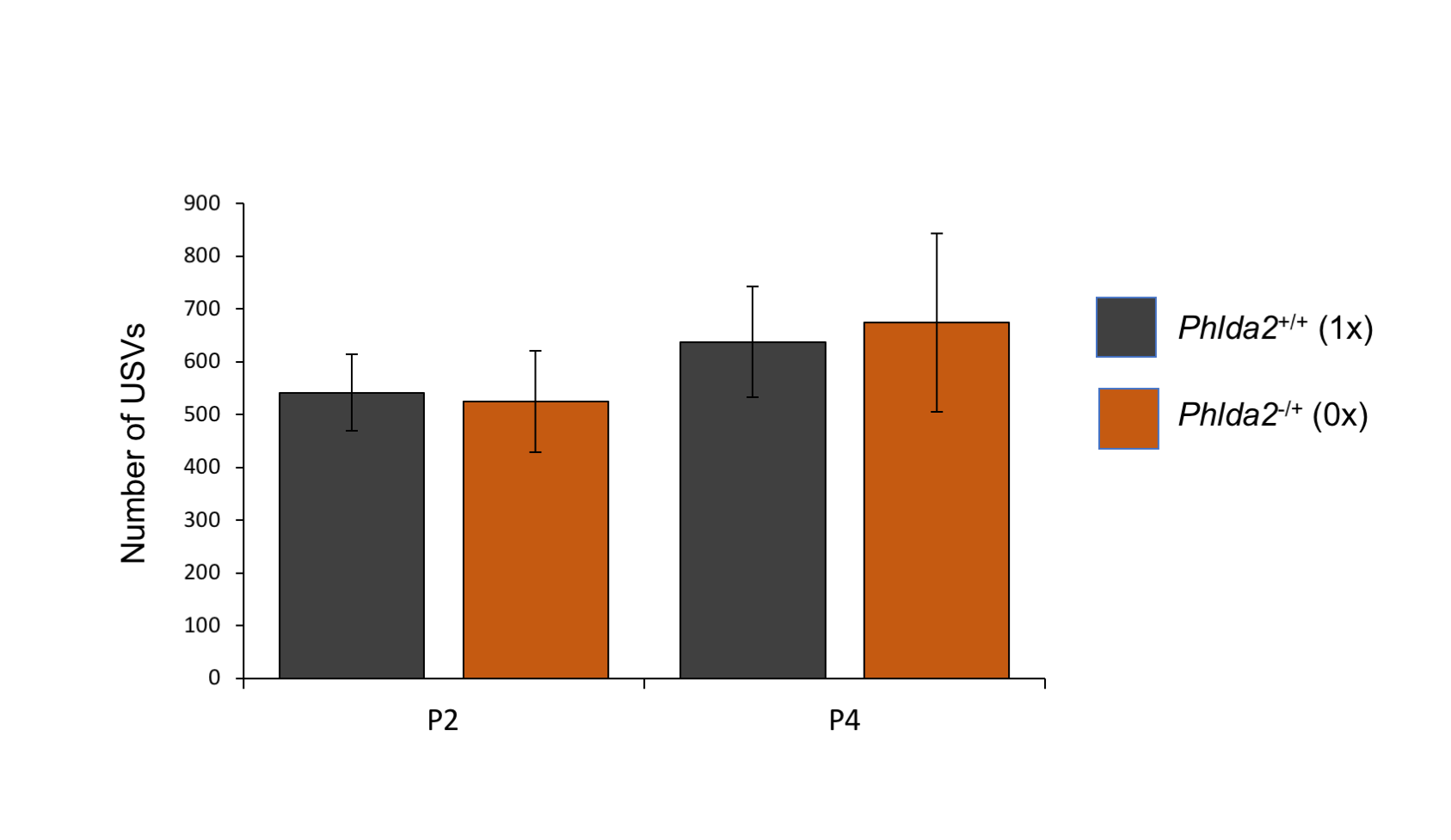

Supplement: S4 Fig — Wild-type pups were generated by mating wild-type, virgin females, aged 5–7 weeks, with wild-type, experienced studs (n = 4). Phlda2−/+ pups were generated by mating homozygous Phlda2−/− virgin females aged 5–7 weeks with wild-type, experienced studs (n = 4). Pup USVs were recorded using Avisoft-UltraSoundGate 116Hb (Avisoft Bioacoustics e.K., Germany) over 180 seconds on P2 and P4. Numerical data can be found at https://osf.io/543jg/ “RAW NUMERICAL DATA.xlsx”, Sheets labelled USV. KO, knockout; P, postnatal day; USV, ultrasonic vocalisation. (TIFF) [file pbio.2006599.s007.tiff]
